# Using Pointwise Mutual Information for Breast Cancer Health Disparities Research With SEER-Medicare Claims

**DOI:** 10.5964/meth.8535

**Published:** 2023-03-31

**Authors:** Brian L. Egleston, Ashis Kumar Chanda, Tian Bai, Carolyn Y. Fang, Richard J. Bleicher, Slobodan Vucetic

**Affiliations:** [1] Biostatistics and Bioinformatics Facility, Fox Chase Cancer Center, Temple University Health System, Philadelphia, PA, USA; [2] Department of Computer and Information Sciences, Temple University, Philadelphia, PA, USA; [3] Cancer Prevention and Control, Fox Chase Cancer Center, Temple University Health System, Philadelphia, PA, USA; [4] Department of Surgical Oncology, Fox Chase Cancer Center, Temple University Health System, Philadelphia, PA, USA

**Keywords:** SEER-Medicare claims, machine learning, pointwise mutual information, breast cancer, health disparities

## Abstract

Identification of procedures using International Classification of Diseases or Healthcare Common Procedure Coding System codes is challenging when conducting medical claims research. We demonstrate how Pointwise Mutual Information can be used to find associated codes. We apply the method to an investigation of racial differences in breast cancer outcomes. We used Surveillance Epidemiology and End Results (SEER) data linked to Medicare claims. We identified treatment using two methods. First, we used previously published definitions. Second, we augmented definitions using codes empirically identified by the Pointwise Mutual Information statistic. Similar to previous findings, we found that presentation differences between Black and White women closed much of the estimated survival curve gap. However, we found that survival disparities were completely eliminated with the augmented treatment definitions. We were able to control for a wider range of treatment patterns that might affect survival differences between Black and White women with breast cancer.

A challenge during the design of studies using medical claims is identification of treatments. This is a nontrivial problem because claims are designed for billing purposes and are only a proxy for patients’ actual treatments. There are thousands of International Classification of Diseases (ICD)-9, ICD-10, and Common Procedural Terminology (CPT) codes in use that identify diagnoses and procedures in medical claims, those codes are updated regularly, and there are numerous ways to encode patients’ conditions and treatments. Medicare incorporates CPT codes into Healthcare Common Procedure Coding System (HCPCS) codes.

In practice, designing rules that identify treatments in Medicare data is a time consuming process based on study of claims and codes, clinical reasoning, and scientific evidence. Miller et al. (2008, 2009), for example, developed an algorithm for identifying laparoscopic surgery among kidney cancer cases before claims codes for laparoscopic surgery were well developed. While such algorithms are useful for others pursuing similar investigations (Smaldone et al., 2012), there may still be substantial mismatch between treatment identified by the SEER cancer registry and treatment identified through Medicare claims. Noone et al. (2016) suggested that Medicare claims should be used to supplement SEER treatment data, as claims are more comprehensive and reliable. Indeed, Bleicher et al. (2012) found substantial mismatch between SEER listed treatments and Medicare claims identified treatments. Hence, regardless of their best efforts, investigators may still find challenging the process of identifying combinations of codes that identify specific treatments. Enhanced methods to efficiently identify relevant codes are needed.

Informed by recent advances in natural language processing, we adapted machine learning algorithms (Levy & Goldberg, 2014a, 2014b) to find vector representations of diagnosis and procedure codes from Medicare claims data, in which related codes that co-occur together or occur in the same contexts or neighborhoods are clustered together. Given an initial set of codes an investigator believes are relevant for identifying a treatment, our method will automatically find related codes. The algorithm is generalizable to changes in codes, such as recent transitions from ICD-9 to ICD-10 codes. In this paper, we document a software assistant that can be used to identify related codes.

We demonstrate the algorithm using a SEER-Medicare breast cancer example. We reproduced, but with more contemporary data, the work of Silber et al. (2013) who found that survival differences between Black and White women in the United States could largely be explained by differences in cancer presentation at diagnosis. That is, while Black women and White women with breast cancer have sizable survival differences, the differences were reduced after controlling for non-cancer comorbidities and severity of disease, such as tumor stage, grade, and lymph node involvement. Still, Silber et al. (2013) found that there were some residual survival differences between Black and White women, even after further controlling for the type of cancer treatment received. We examined whether identifying Medicare treatment codes using our software assistant could possibly better control for confounding when examining racial demographic differences.

## Method

### Participants

We used Surveillance Epidemiology and End Results (SEER) data linked to Medicare claims. SEER is maintained by the National Cancer Institute and has long-term data on tumor characteristics and demographics information about incident cancers for over 14% of the United States (https://seer.cancer.gov/registries/). Expansion since 2000 has resulted in more recent data capturing over 28% of the US population. Medicare covers almost all individuals over 65 years old in SEER. Fee-for-service claims from Medicare part A and part B provide a thorough record of treatments and services obtained before and after cancer diagnosis.

We emulated the same exclusion criteria and methods detailed in the supplement of Silber et al. (2013). We primarily examined cases diagnosed from 1992 to 2005 to largely replicate the sample of Silber et al. (2013) which examined cases through 2005. Since we had additional years of data, we repeated the analyses with cases diagnosed 2006 through 2013 and claims through 2014. We restricted our breast cancer case sample to individuals with Medicare Parts A and B over the age of 66. Those with managed care contracts were excluded due to a lack of claims.

We used propensity score matching to match every Black woman to one White woman using sets of potentially confounding variables that mimic those used by Silber et al. (2013). We matched first on demographics, second on demographics and clinical presentation variables, and third on demographic, clinical presentation, and treatment variables. Silber et al. (2013) used this strategy to show that much of the survival differences between Black and White women largely disappeared after controlling for clinical presentation.

### Instruments

Demographic variables included age, entered into the propensity score model via restricted cubic splines (Harrell, 2001, Ch. 2), and year of diagnosis and SEER registry, entered as categorical variables. Clinical presentation included tumor size (categorical with centimeter increments to ≥ 4 centimeters and a missing indicator), estrogen receptor positivity (ER+), progesterone receptor positivity (PR+), stage of cancer (Categorical I–IV, unknown), grade (five categories including missing) and 25 comorbidities as detailed in the tables. Many of the comorbidities used corresponded to those in the Charlson Comorbidity Index (Charlson et al., 1987).

Treatment included number of nodes removed and positive, entered via restricted cubic splines with four knots, mastectomy, breast conserving therapy, radiation, surgery, chemotherapy, and particularly whether the chemotherapies were doxorubicin or taxanes. We included all two-way, three-way, and four-way treatment interactions in the propensity score model. We did not adjust for neighborhood level income or education variables, as Silber et al. (2013) did not include those in primary analyses.

### Procedure

We identified treatment using two methods. First, we used the ICD-9 and CPT definitions of Silber et al. (2013) directly. We searched for chemotherapy or surgery that occurred within six months of diagnosis, or radiation therapy that occurred within nine months of diagnosis.

The second search method expanded treatment definitions. We developed a machine learning algorithm to identify HCPCS or ICD-9 procedure codes as detailed in Egleston et al. (2021) and Bai et al. (2019) The algorithm allows us to estimate the Pointwise Mutual Information (PMI) statistic that characterizes the strength of relationship between two HCPCS or ICD-9 codes in a Medicare claim (Turney & Pantel, 2010). The PMI relates the joint probability that two codes will be observed in the same claim divided by the probability that the codes will be observed under independence. Software can be accessed at the Supplementary Materials section.

Before presenting the software assistant that implements the algorithm, we define PMI mathematically. Let *C* represent a multinomial random variable denoting the HCPCS or ICD-9 value of an input code of interest, such as one of the breast cancer procedure codes identified by Silber et al. (2013). Let *C*′ be a similar multinomial variable representing codes in the same SEER-Medicare line of a claim of *C* (i.e., close to *C*). We assume that the code at each position in the database is an independent and identically distributed variable whether when considered as an input code (*C*), or a potential claim neighboring code (*C*′). Let subscripts *i* and *j* ∈ {1…*K*} (i.e., *C_i_* and $C_{j}^{\prime }$) be index positions of the codes for a total of *K* codes in the dataset. *K* represents the total number of codes used in the database, not the number of unique values. Let *D_ij_* be a random variable that takes the value 1 if the rule for determining sufficiently close is met for *C_i_* and $C_{j}^{\prime }$, 0 otherwise. The PMI is the log of the probability that two codes are in neighborhoods of each other conditional on being in the set of codes in neighborhoods of each other (i.e., the set in which *D*_*ij*_ = 1), divided by the probability that the two codes are independent conditional upon being in the set in which *D*_*ij*_ = 1. By Bayes’ theorem, this is also equivalent to the log of the conditional probability that *C_i_* is observed conditional on observing $C_{j}^{\prime }$ and meeting the rule *D*_*ij*_ = 1 over the conditional probability of observing *C_i_* given meeting the rule. Formally, the PMI is defined as follows.

$$\text{PMI}=\log\left\{\frac{P\left(C_{i}=c,C_{j}^{\prime }=c^{\prime }|D_{ij}=1\right)}{P\left(C_{i}=c|D_{ij}=1\right)}\right\}\;\text{=log}\left\{\frac{P\left(C_{i}=c|C_{j}^{\prime }=c^{\prime },D_{ij}=1\right)}{P\left(C_{i}=c|D_{ij}=1\right)}\right\}$$

Under independence, $P\left(C_{i}=c|C_{j}^{\prime }=c^{\prime },D_{ij}=1\right)$ would be equal to *P*(*C_i_* = *c*|*D_ij_* = 1), so PMI = log(1) = 0. If the two codes are commonly observed together, then $P\left(C_{i}=c|C_{j}^{\prime }=c^{\prime },D_{ij}=1\right)>P\left(C_{i}=c|D_{ij}=1\right)$ and the ratio will be greater than one, so on the log scale, PMI > 0. One can tokenize the data and then use counts within the tokenized data to estimate the PMI via the component numerators and denominators, or use a logistic regression model detailed in Bai et al. (2019) and Egleston et al. (2021).

**Figure 1:**
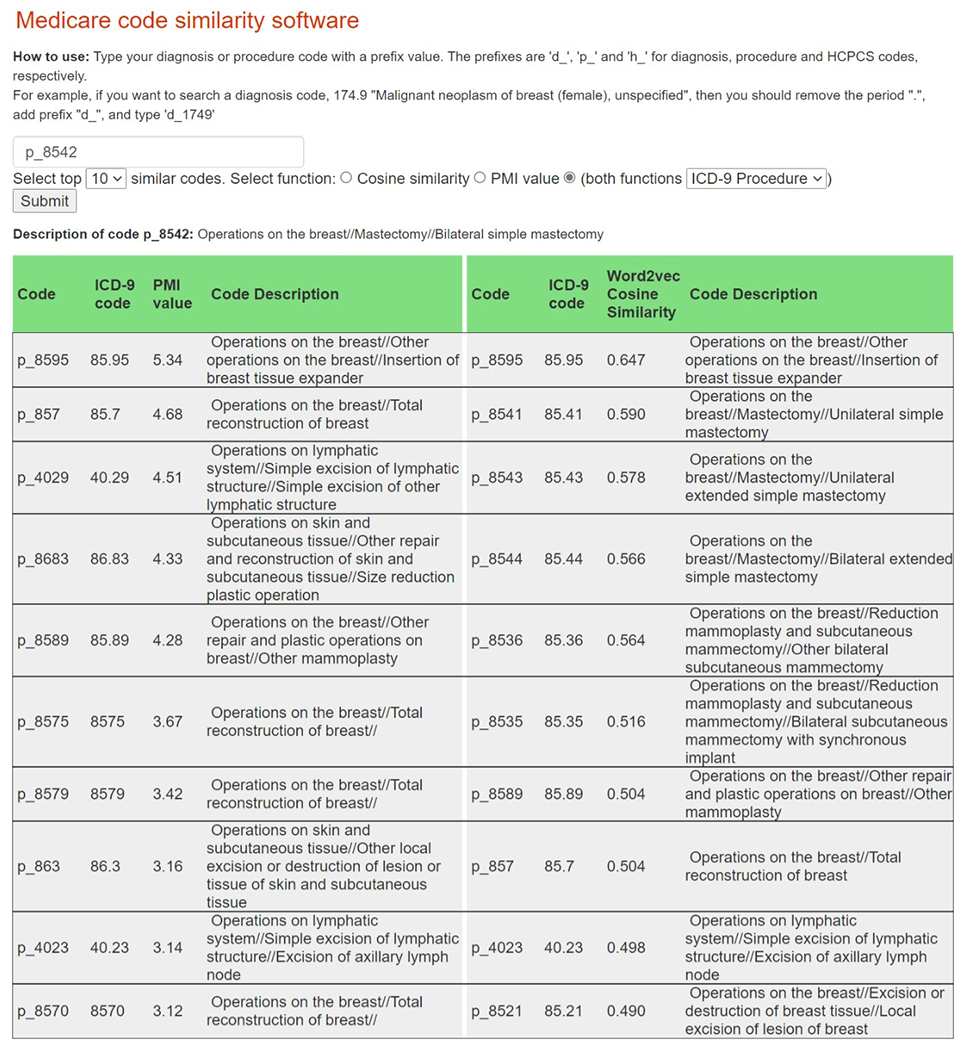
Depiction of Our Software That Can be Used to Search for Related Billing Codes

Our programs calculate the PMI and cosine similarity statistics for comparing two codes in claims data. The algorithms can be tested using one’s own data or synthetic Medicare claims (Center for Medicare Medicaid Services., 2021). In Figure 1, we demonstrate the assistant interface. The software estimates the PMI using code counts of tokenized data and uses the word2vec method found in the python package Gensim (Řehůřek & Sojka, 2010) to find vector representations of codes based on word2vec embeddings (Bai et al., 2019). These vectors are then used to estimate the cosine similarity statistics. In Bai et al. (2019) we previously validated the methods using SEER-Medicare data in which we compared our empirically found codes to those from a clinical paper in which the expert curated codes were published in an appendix (Bleicher et al., 2012). We found that the empirical method identified many of the same codes, but also found three codes that were not listed in the curated set.

In the SEER-Medicare breast cancer data used for this project, we had 67,332,516 lines of claims, 240,150,032 codes, and 36,566 unique ICD-9 and HCPCS code values. Codes were used an average of 6,567 times (*SD* = 118,945). In estimation (i.e., “training”), we excluded infrequent codes used fewer than 50 times in the claims to reduce the computational burden due to high dimensional matrices. This removed 21,215 unique values, but only 218,341 codes from the total (218,341/240,150,032 = 0.1% of total). The codes of most interest were used much more than 50 times. The ICD-9 Code 85.95 (“Operations on the breast//Other operations on the breast//Insertion of breast tissue expander”), for example, was used 7,197 times in the dataset. After removing infrequent codes, each line of a claim had 239,931,691/67,332,516 = 3.56 codes on average. We considered claims to be close to each other, and thus possibly related, if they were on the same line of the claim. This gives approximately 3.56 choose 2 times 67,332,516 = 306,820,808 pairings of codes when not considering order.

After using our programs to estimate the PMI and cosine similarity statistics, one inputs a SEER-Medicare ICD or HCPCS code, and then the related codes with the top PMIs will be displayed. For the time period of our current work, ICD-9 codes were in common use; the assistant will also work with ICD-10 codes. We show an example for an Input Code 85.42, which indicates bilateral simple mastectomy. The ICD-9 Code 85.95 had the highest PMI of 5.34. The assistant will also display related codes with the largest cosine similarity statistics (Huang, 2008). In our case, 85.95 also had the highest word2vec cosine similarity of 0.647.

For each code that Silber et al. (2013) identified, we searched for ICD-9 procedure and HCPCS codes with the largest PMI similarities and used the results to augment breast conserving therapy, mastectomy, and radiation definitions. For chemotherapy receipt in general, as opposed to specific types of chemotherapy, we repeated the process, but only used CPT codes as Silber et al. (2013) had. Then, in an augmented analysis, if a case had a code for mastectomy using either Silber et al. (2013)’s definitions or the augmented definition, we classified that case as having a mastectomy. We did not augment Silber et al. (2013)’s definitions of taxane or doxorubicin chemotherapy as the codes for these are very specific. For surgery, we used the most extensive treatment received in six months. If a woman received breast conserving therapy followed by mastectomy, then we deterministically coded surgery as mastectomy. This algorithm acknowledges that many women may have multiple procedures due to reasons such as positive surgical margins on the first lumpectomy.

A rationale for using expanded and empirically derived definitions to capture treatment is that there is heterogeneity in the codes providers use for reimbursement. For example, a lumpectomy could be billed as an excisional biopsy. By deriving an empirical method of finding treatment codes, we may better identify novel or unusual billing patterns. From a causal inference perspective, the use of empirically derived coding schemes could provide better control of potential confounders. We only controlled for the traditionally identified treatment variables or the augmented variables separately, not together.

### Data Analysis

After forming the matched sample, we examined overall survival using Cox Proportional Hazards regressions and breast cancer specific survival using Fine and Gray (1999) proportional hazards regressions.

## Results

There were 7,753 Black women in our sample from 1992–2005, and 6,186 from 2006–2013. Supplemental Figure 1 (see Supplementary Materials) details our inclusions and exclusions. In Table 1, we present the comparison of the Black women with selected variables from each of the four matched groups. The matching effectively balanced characteristics among the study arms. Supplemental Table 1 (see Supplementary Materials) presents the full set of characteristics of Black and White women in our four matched samples for the time period 1992–2005. Supplemental Table 2 (see Supplementary Materials) presents analogous tables for 2006–2013. Augmented definitions seem to shift many into the mastectomy group, and diminish the proportion in the augmented breast conserving therapy group.

Sample characteristics were similar to those of Silber et al. (2013), but there were small differences. For example, Silber et al. (2013) reported 29.52% of Black women had Grade 2 disease, while we found 31.2%. Our sample size was also a bit larger; Silber et al. (2013) had a sample of 7,375. The comparisons suggest that we well approximated the previously reported sample, even if we did not exactly replicate it. Of note, Silber et al. (2013) likely obtained an earlier version of the SEER-Medicare dataset which we did not access.

In Supplemental Table 3 (see the Supplementary Materials section), we present the mapping of Silber’s codes to the top related codes based on PMI. In creating our augmented treatment definitions, we remained agnostic as to whether the codes truly defined the four therapies of most interest: breast conserving therapy (BCT), mastectomy, radiation therapy, or chemotherapy. Hence, we used ICD-9 code 85.95 in the augmented mastectomy definition, even though it represents “Operations on the breast//Other operations on the breast//Insertion of breast tissue expander”. Although this was not directly related to mastectomy, it was the ICD-9 code most likely to be found in the same claim with ICD-9 procedure code 85.42 which indicates “bilateral simple mastectomy.” It may be reasonable to assume for purposes of controlling for confounders that a woman with breast cancer who has such a code might likely have had a mastectomy.

**Figure 2:**
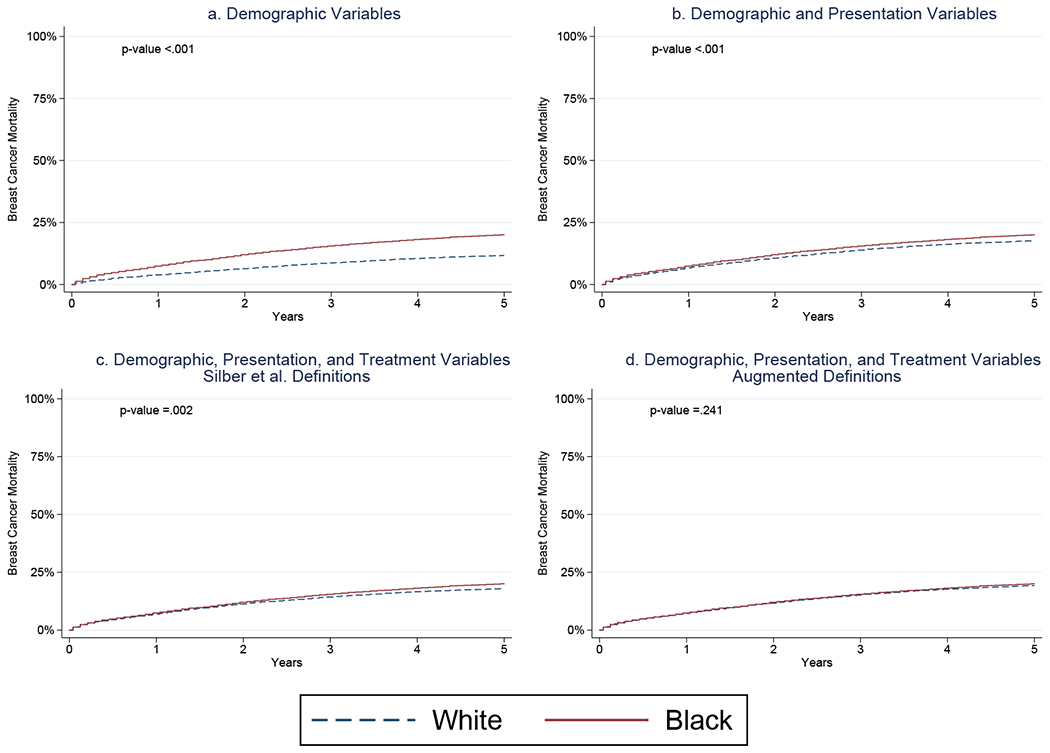
Breast Cancer Mortality Estimates Under Various Matching Schemes for Cases Diagnosed, 1992–2005

In Figure 2, we present cumulative incidence curves of breast cancer specific mortality within five years of diagnosis. Similarly to Silber et al. (2013), we found that Black women had higher mortality than White women after matching only on a limited number of demographic variables available in the SEER data (Figure 2a). After matching on demographic and presentation variables, much of the survival difference between Black and White women was largely attenuated (Figure 2b), but the difference was still statistically significant. However, our curves may suggest a greater narrowing of differences when adjusting for presentation variables than Silber et al. (2013) did. After further adjusting for treatments using Silber et al. (2013)’s definitions (Figure 2c), the difference in survival became less marked. When using our augmented definitions, the curves overlap, and the difference in the cumulative incidence of breast cancer death between Black and White women is largely eliminated (Figure 2d).

**Figure 3:**
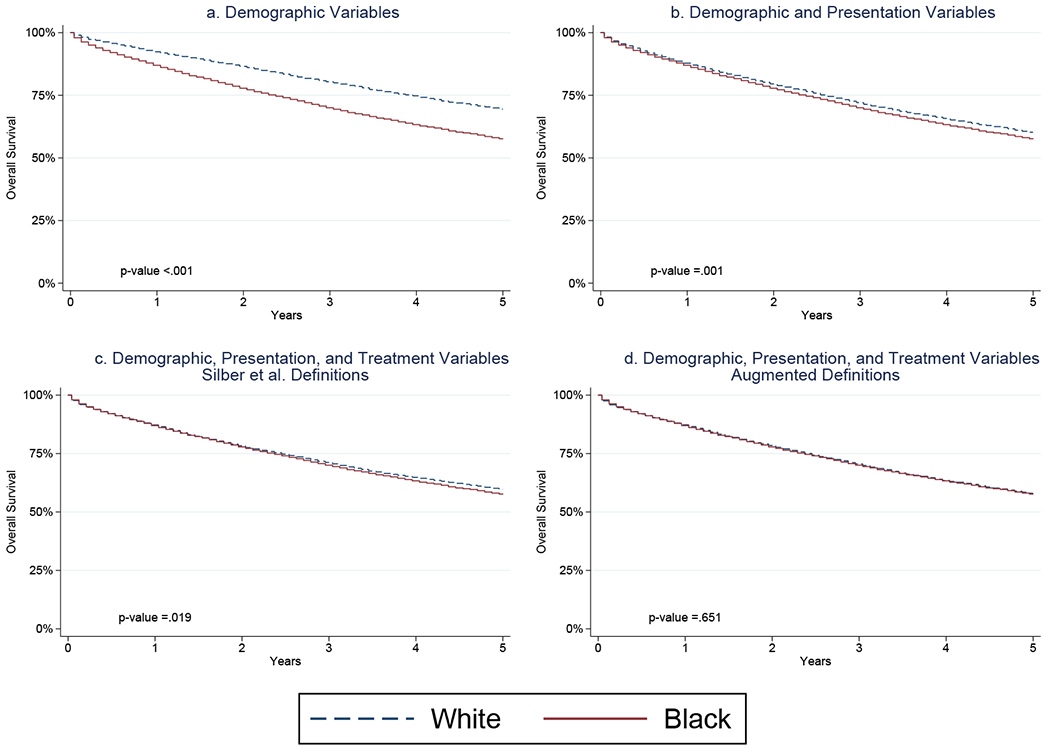
Overall Survival Under Various Matching Schemes for Cases Diagnoses, 1992–2005

Our overall survival findings showed similar congruence with those of Silber et al. (2013). Racial survival differences still persist after matching on demographic variables (Figure 3a). Again, the overall survival differences persist but are greatly reduced after controlling for presentation (Figure 3b) and treatment variables (Figure 3c). After controlling for the augmented treatment differences, the survival curves for White and Black women are almost completely overlapping (Figure 3d). Hence, using augmented treatment definition data pre-2006 suggests that the residual effect of race on survival after further controlling for presentation and treatment has been eliminated. Overall, these demonstrate substantial differences from the pre-2006 era reported by Silber et al. (2013).

In Supplementary Figures 2 and 3 (see the Supplementary Materials section), we replicate the analyses, but use 2006–2013 data, which is for a later time period than reported by Silber et al. (2013). We find differences from the earlier period. For breast cancer specific survival, we found that differences were largely eliminated after controlling for presentation variables (see Supplemental Figure 2b in the Supplementary Materials section). The lack of difference similarly persisted after controlling for treatment variables (see Supplemental Figures 2c and 2d in the Supplementary Materials section). The pattern is similar for overall survival.

## Discussion

Racial disparities in cancer survival outcomes have been of interest to researchers. Many simply describe the difference without providing sufficient analytic details that could explain causal mechanisms (e.g. Wheeler et al., 2013). Others only have access to a limited number of variables that can be used to control for confounding between Black and White women, such as studies that rely on SEER without linked Medicare data (Aizer et al., 2014; Iqbal et al., 2015). In the statistical causal inference field, many have argued that race should not be studied without consideration of the variables or societal attitudes that can cause differences among racial subgroups (Greiner & Rubin, 2011). Without accounting for potentially confounding variables, examination of racial differences can potentially exacerbate negative attitudes about race and hinder targeted efforts to end discrimination.

By using linked SEER-Medicare data, it is possible to examine confounders and better isolate reasons that racial differences in outcomes persist. The findings of Silber et al. (2013) provided valuable information that much of the difference in racial outcomes in breast cancer could be explained by presentation differences prior to 2006. The clinical stage and aggressiveness of the disease at diagnosis seemed to be driving health disparities. We similarly found that differences were largely attenuated after controlling for presentation variables, and the addition of traditionally derived treatment variables did not further change relationships of race with outcomes. By contrast, we found that the addition of augmented treatment variables closed the gap between the survival curves and indicated that there were no differences between groups after controlling for a more expansive list of HCPCS and ICD-9 codes.

One major difference between our work and others’ work is that we did not screen our codes to determine if the related codes found during the augmentation process were truly reflective of the treatment categories into which they were grouped. Our algorithm would hence not be appropriate for investigations of treatment effects in which a treatment must be well defined, such as that undertaken by Petito et al. (2020). Our approach seems most appropriate in studies that seek to remove the confounding effect of variables. Indeed, treatment effects were not a primary interest of our paper, but controlling for the impact that they can have on inferences about how race impacts breast cancer differences was a goal. Hence, while our augmented treatment groups may not be interpretable as internally consistent treatment groups, they did capture broad ways in which the nomenclature concerning treatment can vary within claims.

Our method also involves the combination of subject matter expert evaluation of relevant claims codes with a more algorithmic approach in grouping codes. Often, the identification of relevant confounders in high dimensional data is seen as one of either using subject matter experts to narrow down the codes into relevant groupings, or using machine learning or Bayesian approaches to empirically select relevant codes (e.g. Spertus & Normand, 2018). Our approach combines clinical expertise with an empirical approach to categorize codes into intervention groups.

One limitation to our work is that the treatment groups we created were not necessarily meaningful. That is, some ancillary treatments, such as reconstruction, were grouped with mastectomy codes. Hence, our method might not be appropriate for investigating intervention effects in which the intervention itself is of interest. While we did provide preliminary validation of our empirically derived codes compared to human expert curation (Bai et al., 2019), additional studies are needed to more rigorously validate our algorithms.

The fact that the curves narrowed substantially after creating the augmented definitions suggests that there could have been racial differences in treatments chosen or how procedures were coded for billing purposes. Another possibility is that there were racial differences in the sequence of therapy. Many women who choose breast conserving therapy may need repeated operations due to findings such as positive surgical margins (Morrow et al., 2009). It is possible that the algorithmic approach was better at classifying treatments into groups that better captured such practice patterns. Future research can investigate why coding algorithms may differ between Black and White women and hence confound any differences in outcomes by race. It could be that there are true racial differences in treatments received, or it could be that similar treatments tend to be coded for billing and claims purposes differently between Black and White women.

We also found that the racial differences appeared diminished after controlling for presentation in the more recent data from 2006 to 2013. Although we did not formally test for temporal differences, many new treatments have been approved since 2006 that could potentially have affected survival, particularly for those with advanced disease (Cortazar et al., 2012). As this included a population eligible for Medicare, the introduction of U.S. prescription drug coverage through Medicare Part D in 2007 might also have improved access to prescription therapies in the later period.

Our method is similar to emerging hybrid artificial intelligence (AI) approaches that augment, rather than replace, human expertise with machine learning (Zheng et al., 2017). We used expert derived codes augmented by empirically found codes to better capture potential confounding between disease groups. In the case of SEER-Medicare data, hybrid AI is useful due to the large number of ICD-9/10 and HCPCS codes. There are often multiple ways to code the same event for Medicare reimbursement purposes. For example, excisional biopsy and lumpectomy may be used to describe the same tumor removal procedure. In such cases, hybrid-AI might assist researchers in identifying patterns of claims that reflect equivalent procedures. In the context of propensity score analyses, hybrid-AI can help expand the number of confounders used in adjustment. Besides claims data, hybrid-AI has been used in propensity score based analyses of geographic information system (GIS) data (Monlezun et al., 2021).

In conclusion, we proposed an application of a machine learning algorithm that uses the pointwise mutual information statistic to identify related codes when using Medicare claims data. By using this algorithm, we were able to control for a wider range of treatment patterns that potentially differentially affect survival differences between Black to White women with breast cancer. Similar to previous estimates, we have found that presentation differences between Black and White women closed much of the estimated survival curve gap. However, it is possible that treatment differences identified by our application could further explain racial differences in outcomes. Future work will be necessary to better explore the specific differences that may be contributing to health disparities.

## Supplementary Material

Supplementary Material

## Figures and Tables

**Table 1:** Comparison of Selected Variables Used for Matching.

Variable	Black	Demographic Match White	Plus Presentation White	Plus Treatment White	Plus Treatment Augmented White
**Demographic Variables: *M (SD)***					
Age	75.86 (7.07)	75.82 (7.00)	75.78 (6.99)	75.92 (7.07)	75.89 (7.12)
Size (mm)	27.67 (26.59)	20.99 (21.14)	26.51 (26.64)	26.61 (28.88)	26.85 (23.32)

**Presentation Variables: Number (%)**					
**AJCC Stage**					
Stage I	3015 (38.9%)	4221 (54.4%)	3015 (38.9%)	3050 (39.3%)	3012 (38.8%)
Stage II	3085 (39.8%)	2593 (33.4%)	3098 (40.0%)	3066 (39.5%)	3067 (39.6%)
Stage III	897 (11.6%)	534 (6.9%)	887 (11.4%)	875 (11.3%)	902 (11.6%)
Stage IV	756 (9.8%)	405 (5.2%)	753 (9.7%)	762 (9.8%)	772 (10.0%)
**Tumor Grade**					
Grade I	1037 (13.4%)	1508 (19.5%)	1051 (13.6%)	1053 (13.6%)	997 (12.9%)
Grade II	2419 (31.2%)	2971 (38.3%)	2435 (31.4%)	2397 (30.9%)	2376 (30.6%)
Grade III	2645 (34.1%)	1992 (25.7%)	2584 (33.3%)	2614 (33.7%)	2695 (34.8%)
Grade IV	143 (1.8%)	92 (1.2%)	114 (1.5%)	151 (1.9%)	153 (2.0%)
Unknown	1509 (19.5%)	1190 (15.3%)	1569 (20.2%)	1538 (19.8%)	1532 (19.8%)

**Treatment Variables, Number (%)**					
**BCT**					
No BCT	6080 (78.4%)	5985 (77.2%)	6199 (80.0%)	6044 (78.0%)	6130 (79.1%)
BCT	1673 (21.6%)	1768 (22.8%)	1554 (20.0%)	1709 (22.0%)	1623 (20.9%)
**Mastectomy**					
No Mastectomy	2684 (34.6%)	2254 (29.1%)	2293 (29.6%)	2712 (35.0%)	2575 (33.2%)
Mastectomy	5069 (65.4%)	5499 (70.9%)	5460 (70.4%)	5041 (65.0%)	5178 (66.8%)
**Radiation**					
No	4711 (60.8%)	4128 (53.2%)	4465 (57.6%)	4692 (60.5%)	4597 (59.3%)
Yes	3042 (39.2%)	3625 (46.8%)	3288 (42.4%)	3061 (39.5%)	3156 (40.7%)
**Chemotherapy**					
No	5795 (74.7%)	6165 (79.5%)	5709 (73.6%)	5754 (74.2%)	5758 (74.3%)
Yes	1958 (25.3%)	1588 (20.5%)	2044 (26.4%)	1999 (25.8%)	1995 (25.7%)

**Augmented Treatment Variables, Number (%)**					
**BCT** ^a^					
No BCT	7145 (92.2%)	7276 (93.8%)	7249 (93.5%)	7224 (93.2%)	7149 (92.2%)
BCT	608 (7.8%)	477 (6.2%)	504 (6.5%)	529 (6.8%)	604 (7.8%)
**Mastectomy** ^a^					
No Mastectomy	1477 (19.1%)	883 (11.4%)	1137 (14.7%)	1390 (17.9%)	1461 (18.8%)
Mastectomy	6276 (80.9%)	6870 (88.6%)	6616 (85.3%)	6363 (82.1%)	6292 (81.2%)
**Radiation** ^a^					
No	4604 (59.4%)	4046 (52.2%)	4356 (56.2%)	4579 (59.1%)	4513 (58.2%)
Yes	3149 (40.6%)	3707 (47.8%)	3397 (43.8%)	3174 (40.9%)	3240 (41.8%)
**Chemo** ^a^					
No	5717 (73.7%)	6019 (77.6%)	5577 (71.9%)	5619 (72.5%)	5632 (72.6%)
Yes	2036 (26.3%)	1734 (22.4%)	2176 (28.1%)	2134 (27.5%)	2121 (27.4%)

*Note*. Internal lines differentiate variables that were and were not matched upon within columns, and we included traditional and augmented treatments separately;

BCT = Breast conserving therapy.

a Augmented treatment definition.

## Data Availability

The SEER-Medicare data used is publicly available from the U.S. National Cancer Institute. We also provide synthetic Medicare claims data, freely available in the Supplementary Materials.

## Supplementary Materials

The supplementary materials provided are the MedCS: Medicare Code Similarity software (for Windows 7 or above) in Egleston et al. (2021), and the Supplementary Tables and Figures in Egleston et al. (2023). They can be accessed in the Index of Supplementary Materials below.
