# Supplementary material for: Using Pointwise Mutual Information for Breast Cancer Health Disparities Research With SEER-Medicare Claims
Source: Methodology (Gott). Author manuscript; Available in PMC 2023 Apr 21. (PMC10121207; doi:10.5964/meth.8535)
Supplement: Supplementary Material [file NIHMS1888195-supplement-Supplementary_Material.pdf]

Supplementary material for the article: Brian L. Egleston, Ashis Kumar Chanda, Tian Bai, Carolyn Y. Fang, Richard J. Bleicher, Slobodan Vucetic, "Using pointwise mutual information in breast cancer health disparities research with SEER-Medicare claims," ***Methodology.***

Supplemental Material Table of Contents

Page 2. Supplemental Figure 1: Flowchart of inclusions and exclusions.
Page 3. Supplemental Figure 2: Breast cancer specific mortality under various matching schemes for cases diagnosed 2006-2013.
Page 4. Supplemental Figure 3: Overall survival estimates under various matching schemes for cases diagnosed 2006-2013.
Page 5. Supplemental Table 1. Demographic, presentation, and treatment characteristics in the 1992-2005 sample after adjusting for the various sets of confounders. *Small cell values are obscured for privacy purposes.

- **a.** Adjusting for Demographic Variables, 1992-2005.
- **b.** Adjusting for Demographic and Presentation Variables, 1992-2005.
- **c.** Adjusting for Demographic, Presentation, and Treatment Variables, 1992-2005.
- **d.** Adjusting for Demographic, Presentation, and Augmented Treatment Variables (a = Augmented Definitions), 1992-2005.

Page 32. Supplemental Table 2. Demographic, presentation, and treatment characteristics in the 2006-2013 sample after adjusting for the various sets of confounders. *Small cell values are obscured for privacy purposes.

- **a.** Adjusting for Demographic Variables, 2006-2013.
- **b.** Adjusting for Demographic and Presentation Variables, 2006-2013.
- **c.** Adjusting for Demographic, Presentation, and Treatment Variables, 2006-2013.
- **d.** Adjusting for Demographic, Presentation, and Augmented Treatment Variables (a = Augmented Definitions), 2006-2013.

Page 58. Supplemental Table 3. Mapping of Silber et al. (2013) codes to the code with the highest Pointwise Mutual Information (PMI) statistic.

Acronym: BCT = Breast Conserving Therapy

Supplemental Figure 1.

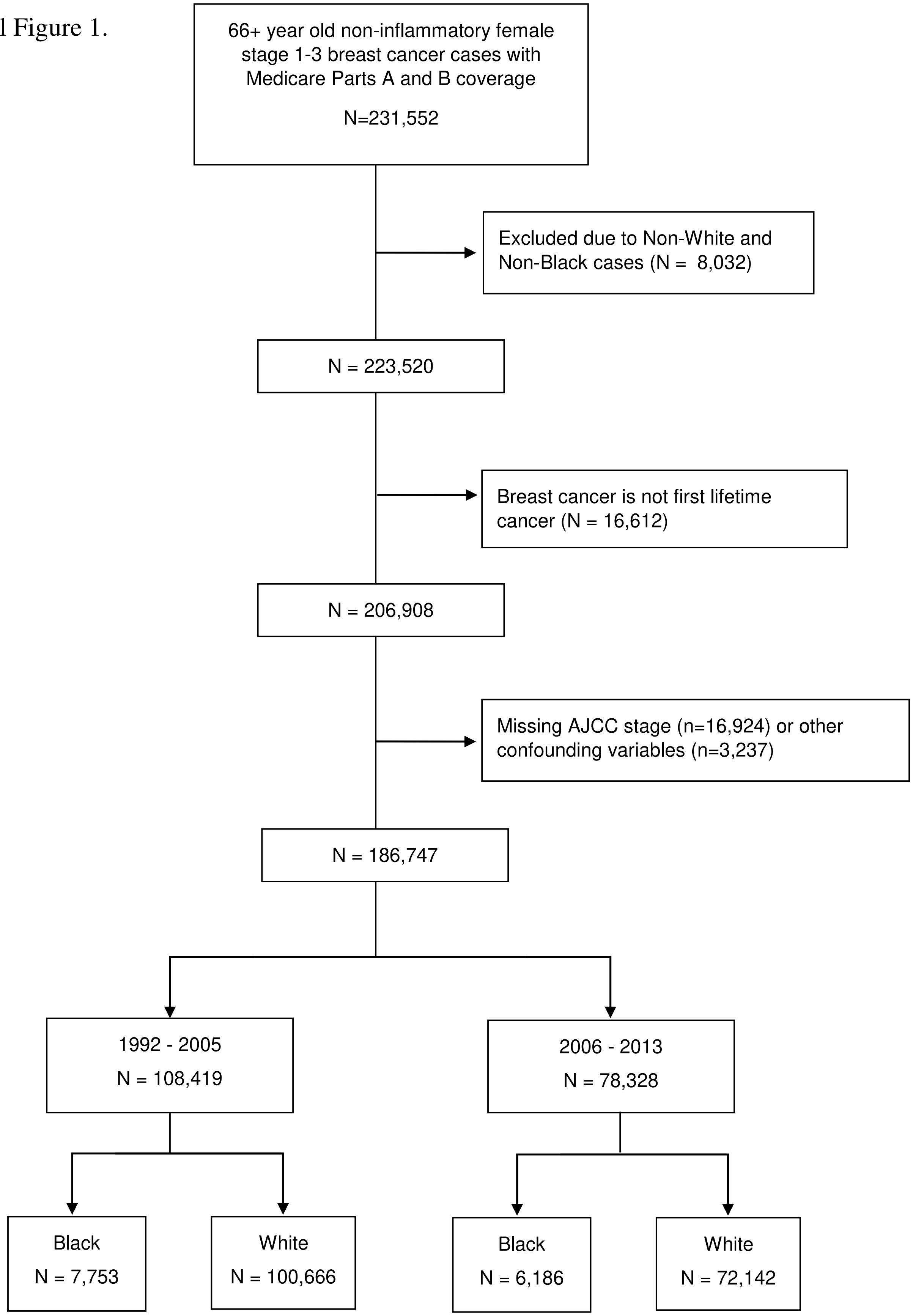

Supplemental Figure 2: Breast cancer specific mortality under various definitions, 2006-2013.

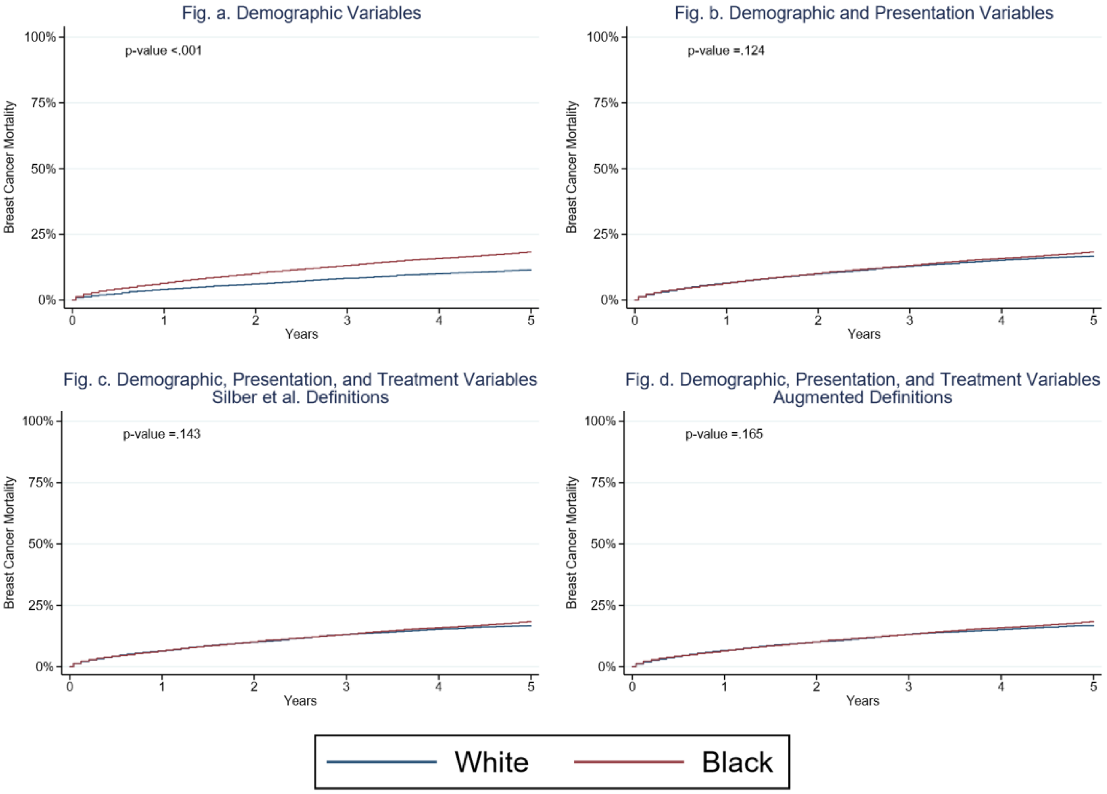

Supplemental Figure 3: Overall survival estimates under various matching schemes for cases diagnoses, 2006-2013.

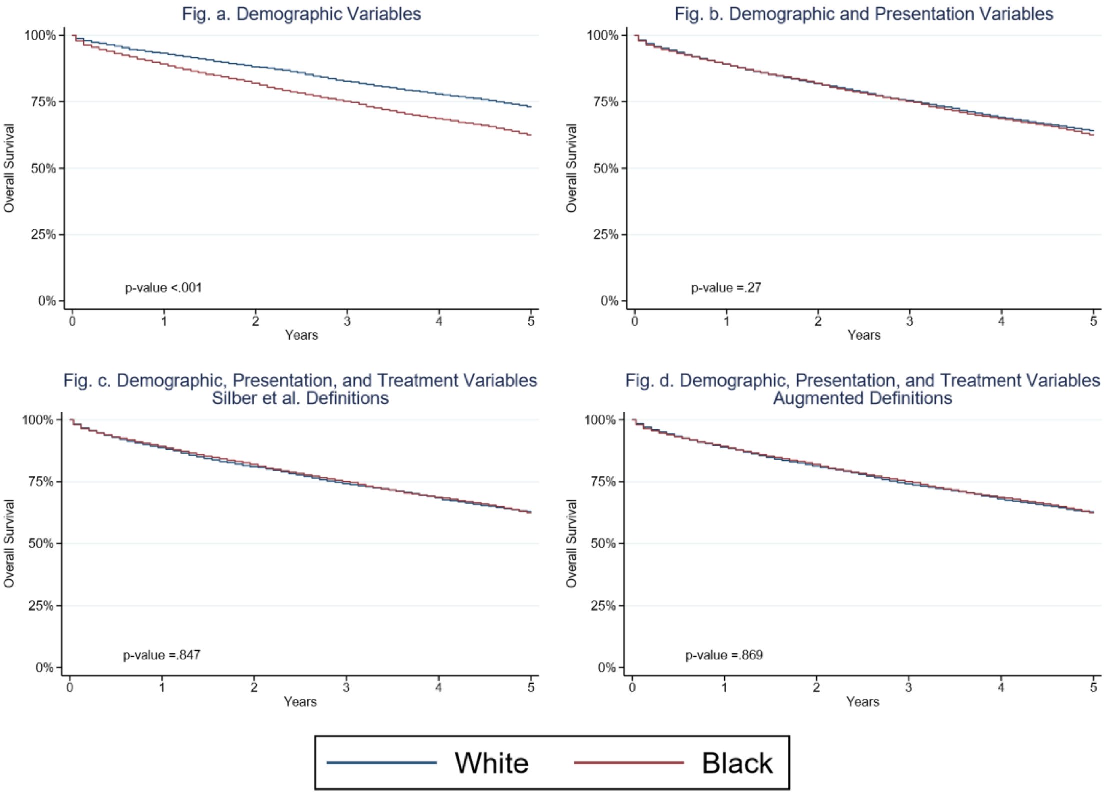

Supplemental Table 1. Characteristics between Black and White women for the period 1992-2005 after adjusting for the various sets of confounders. *Small cell values are obscured for privacy purposes.

Supplemental Table 1a. Controlling for Demographic Variables, 1992-2005

	White (N = 7753)	Black (N = 7753)	p-value
**Age**			0.698
Mean (SD)	75.82 (7.00)	75.86 (7.07)	
Median (Q1, Q3)	75.0 (70.0, 80.0)	75.0 (70.0, 80.0)	
**Size (mm)**			<0.001
Mean (SD)	20.99 (21.14)	27.67 (26.59)	
Median (Q1, Q3)	15.0 (10.0, 25.0)	20.0 (12.0, 35.0)	
**Number of Positive Nodes**			<0.001
Mean (SD)	0.94 (2.91)	1.22 (3.29)	
Median (Q1, Q3)	0.0 (0.0, 0.0)	0.0 (0.0, 1.0)	
**Number of Nodes Examined**			0.044
Mean (SD)	8.26 (7.80)	8.00 (7.93)	
Median (Q1, Q3)	7.0 (1.0, 14.0)	7.0 (0.0, 14.0)	
**Year of Diagnosis**			1.000
1992	383 (4.9%)	381 (4.9%)	
1993	324 (4.2%)	325 (4.2%)	
1994	318 (4.1%)	322 (4.2%)	
1995	321 (4.1%)	327 (4.2%)	
1996	343 (4.4%)	338 (4.4%)	
1997	328 (4.2%)	328 (4.2%)	
1998	325 (4.2%)	329 (4.2%)	
1999	301 (3.9%)	302 (3.9%)	
2000	861 (11.1%)	866 (11.2%)	
2001	824 (10.6%)	825 (10.6%)	
2002	874 (11.3%)	874 (11.3%)	
2003	868 (11.2%)	863 (11.1%)	
2004	834 (10.8%)	835 (10.8%)	
2005	849 (11.0%)	838 (10.8%)	
**Registry**			1.000
Connecticut	325 (4.2%)	326 (4.2%)	
Detroit	1835 (23.7%)	1842 (23.8%)	
Hawaii	* (*)	* (*)	
Iowa	69 (0.9%)	69 (0.9%)	
New Mexico	* (*)	* (*)	
Seattle	106 (1.4%)	104 (1.3%)	
Utah	* (*)	* (*)	
Kentucky	237 (3.1%)	238 (3.1%)	
Louisiana	914 (11.8%)	908 (11.7%)	
New Jersey	805 (10.4%)	800 (10.3%)	
Georgia	1802 (23.2%)	1801 (23.2%)	
California	1622 (20.9%)	1625 (21.0%)	
**AJCC Stage**			<0.001

	White (N = 7753)	Black (N = 7753)	p-value
Stage I	4221 (54.4%)	3015 (38.9%)	
Stage II	2593 (33.4%)	3085 (39.8%)	
Stage III	534 (6.9%)	897 (11.6%)	
Stage IV	405 (5.2%)	756 (9.8%)	
**Tumor Grade**			<0.001
Grade I	1508 (19.5%)	1037 (13.4%)	
Grade II	2971 (38.3%)	2419 (31.2%)	
Grade III	1992 (25.7%)	2645 (34.1%)	
Grade IV	92 (1.2%)	143 (1.8%)	
Missing	1190 (15.3%)	1509 (19.5%)	
**ER Status**			<0.001
Negative	1007 (13.0%)	1577 (20.3%)	
Positive	5148 (66.4%)	4315 (55.7%)	
Missing	1598 (20.6%)	1861 (24.0%)	
**PR Status**			<0.001
Negative	1912 (24.7%)	2412 (31.1%)	
Positive	4137 (53.4%)	3406 (43.9%)	
Missing	1704 (22.0%)	1935 (25.0%)	
**Tumor Size**			<0.001
0-.9cm	1604 (20.7%)	1105 (14.3%)	
1-1.9	2911 (37.5%)	2149 (27.7%)	
2-2.9	1424 (18.4%)	1572 (20.3%)	
3-3.9	640 (8.3%)	855 (11.0%)	
4+cm	894 (11.5%)	1555 (20.1%)	
Missing	280 (3.6%)	517 (6.7%)	
**Congestive Heart Failure**			<0.001
No	6648 (85.7%)	6134 (79.1%)	
Yes	1105 (14.3%)	1619 (20.9%)	
**Past Arrhythmia**			<0.001
No	6870 (88.6%)	6706 (86.5%)	
Yes	883 (11.4%)	1047 (13.5%)	
**Past Myocardial Infarction**			0.561
No	7444 (96.0%)	7458 (96.2%)	
Yes	309 (4.0%)	295 (3.8%)	
**Angina**			0.116
No	7342 (94.7%)	7297 (94.1%)	
Yes	411 (5.3%)	456 (5.9%)	
**Valvular Heart Disease**			0.006
No	6952 (89.7%)	7054 (91.0%)	
Yes	801 (10.3%)	699 (9.0%)	
**Hypertension (uncomplicated)**			<0.001
No	3524 (45.5%)	3258 (42.0%)	
Yes	4229 (54.5%)	4495 (58.0%)	
**Hypertension (complications)**			<0.001
No	6712 (86.6%)	5708 (73.6%)	

	White (N = 7753)	Black (N = 7753)	p-value
Yes	1041 (13.4%)	2045 (26.4%)	
**Diabetes (uncomplicated)**			<0.001
No	6546 (84.4%)	5852 (75.5%)	
Yes	1207 (15.6%)	1901 (24.5%)	
**Diabetes (complications)**			<0.001
No	7353 (94.8%)	6767 (87.3%)	
Yes	400 (5.2%)	986 (12.7%)	
**Kidney Disease**			<0.001
None	7495 (96.7%)	7065 (91.1%)	
Renal Dysfunction	81 (1.0%)	151 (1.9%)	
Renal Failure	177 (2.3%)	537 (6.9%)	
**Liver disease**			0.572
No	7679 (99.0%)	7672 (99.0%)	
Yes	74 (1.0%)	81 (1.0%)	
**Chronic Lung Disease**			0.008
No	6210 (80.1%)	6339 (81.8%)	
Yes	1543 (19.9%)	1414 (18.2%)	
**Dementia**			<0.001
No	7392 (95.3%)	7159 (92.3%)	
Yes	361 (4.7%)	594 (7.7%)	
**Ischemic event**			0.552
No	7550 (97.4%)	7538 (97.2%)	
Yes	203 (2.6%)	215 (2.8%)	
**Stroke**			<0.001
No	7599 (98.0%)	7479 (96.5%)	
Yes	154 (2.0%)	274 (3.5%)	
**Hypothyroidism**			<0.001
No	6065 (78.2%)	6896 (88.9%)	
Yes	1688 (21.8%)	857 (11.1%)	
**Paralysis**			<0.001
No	7566 (97.6%)	7341 (94.7%)	
Yes	187 (2.4%)	412 (5.3%)	
**Collagen Vascular Disease**			0.067
No	7432 (95.9%)	7385 (95.3%)	
Yes	321 (4.1%)	368 (4.7%)	
**Coagulopathy**			0.192
No	7358 (94.9%)	7393 (95.4%)	
Yes	395 (5.1%)	360 (4.6%)	
**Chronic Obstructive Pulmonary Disease**			0.030
No	6182 (79.7%)	6289 (81.1%)	
Yes	1571 (20.3%)	1464 (18.9%)	
**Peptic Ulcer Disease**			0.537
No	7734 (99.8%)	7730 (99.7%)	
Yes	19 (0.2%)	23 (0.3%)	
**Depression**			<0.001

	White (N = 7753)	Black (N = 7753)	p-value
No	7157 (92.3%)	7336 (94.6%)	
Yes	596 (7.7%)	417 (5.4%)	
**Cushing's Disease**			0.705
No	* (100%)	* (100%)	
Yes	* (0%)	* (0%)	
**Graves Disease**			0.655
No	* (100%)	* (100%)	
Yes	* (0%)	* (0%)	
**Post-Inflammatory Pulmonary Fibrosis**			0.067
No	7662 (98.8%)	7685 (99.1%)	
Yes	91 (1.2%)	68 (0.9%)	
**Any Breast Conserving Therapy**			0.066
No BCT	5985 (77.2%)	6080 (78.4%)	
BCT	1768 (22.8%)	1673 (21.6%)	
**Any Mastectomy**			<0.001
No Mastectomy	2254 (29.1%)	2684 (34.6%)	
Mastectomy	5499 (70.9%)	5069 (65.4%)	
**Any Radiation**			<0.001
No	4128 (53.2%)	4711 (60.8%)	
Yes	3625 (46.8%)	3042 (39.2%)	
**Any Chemotherapy**			<0.001
No	6165 (79.5%)	5795 (74.7%)	
Yes	1588 (20.5%)	1958 (25.3%)	
**Any Doxorubicin**			0.002
No	7032 (90.7%)	6917 (89.2%)	
Yes	721 (9.3%)	836 (10.8%)	
**Any Taxane**			0.056
No	7340 (94.7%)	7285 (94.0%)	
Yes	413 (5.3%)	468 (6.0%)	
**Doxorubicin and BCT**			0.864
No	7599 (98.0%)	7596 (98.0%)	
Yes	154 (2.0%)	157 (2.0%)	
**Doxorubicin and Mastectomy**			0.128
No	7220 (93.1%)	7171 (92.5%)	
Yes	533 (6.9%)	582 (7.5%)	
**Doxorubicin and Radiation**			0.387
No	7323 (94.5%)	7298 (94.1%)	
Yes	430 (5.5%)	455 (5.9%)	
**Doxorubicin and Taxane**			0.838
No	7437 (95.9%)	7442 (96.0%)	
Yes	316 (4.1%)	311 (4.0%)	
**Taxane and BCT**			0.882
No	7660 (98.8%)	7662 (98.8%)	
Yes	93 (1.2%)	91 (1.2%)	
**Taxane and Mastectomy**			0.796

	White (N = 7753)	Black (N = 7753)	p-value
No	7478 (96.5%)	7472 (96.4%)	
Yes	275 (3.5%)	281 (3.6%)	
**Taxane and Radiation**			0.791
No	7484 (96.5%)	7490 (96.6%)	
Yes	269 (3.5%)	263 (3.4%)	
**Radiation and BCT**			<0.001
No	6450 (83.2%)	6671 (86.0%)	
Yes	1303 (16.8%)	1082 (14.0%)	
**Radiation and Mastectomy**			<0.001
No	5546 (71.5%)	6024 (77.7%)	
Yes	2207 (28.5%)	1729 (22.3%)	
**Radiation, Doxorubicin, Taxane**			0.040
No	7528 (97.1%)	7569 (97.6%)	
Yes	225 (2.9%)	184 (2.4%)	
**BCT, Doxorubicin, Taxane**			0.859
No	7688 (99.2%)	7690 (99.2%)	
Yes	65 (0.8%)	63 (0.8%)	
**BCT, Doxorubicin, Radiation**			0.487
No	7620 (98.3%)	7631 (98.4%)	
Yes	133 (1.7%)	122 (1.6%)	
**BCT, Taxane, Radiation**			0.497
No	7679 (99.0%)	7687 (99.1%)	
Yes	74 (1.0%)	66 (0.9%)	
**Mastectomy, Doxorubicin, Taxane**			0.155
No	7525 (97.1%)	7554 (97.4%)	
Yes	228 (2.9%)	199 (2.6%)	
**Mastectomy, Doxorubicin, Radiation**			0.831
No	7472 (96.4%)	7467 (96.3%)	
Yes	281 (3.6%)	286 (3.7%)	
**Mastectomy, Taxane, Radiation**			0.295
No	7575 (97.7%)	7594 (97.9%)	
Yes	178 (2.3%)	159 (2.1%)	
**Mastectomy, Doxorubicin, Taxane, Radiation**			0.009
No	7595 (98.0%)	7638 (98.5%)	
Yes	158 (2.0%)	115 (1.5%)	
**BCT, Doxorubicin, Taxane, Radiation**			0.436
No	7696 (99.3%)	7704 (99.4%)	
Yes	57 (0.7%)	49 (0.6%)	
**Any Breast Conserving Therapy (a)**			<0.001
No BCT	7276 (93.8%)	7145 (92.2%)	
BCT	477 (6.2%)	608 (7.8%)	
**Any Mastectomy (a)**			<0.001
No Mastectomy	883 (11.4%)	1477 (19.1%)	
Mastectomy	6870 (88.6%)	6276 (80.9%)	

	White (N = 7753)	Black (N = 7753)	p-value
**Any Radiation (a)**			<0.001
No	4046 (52.2%)	4604 (59.4%)	
Yes	3707 (47.8%)	3149 (40.6%)	
**Any Chemotherapy (a)**			<0.001
No	6019 (77.6%)	5717 (73.7%)	
Yes	1734 (22.4%)	2036 (26.3%)	
**Any Doxorubicin**			0.002
No	7032 (90.7%)	6917 (89.2%)	
Yes	721 (9.3%)	836 (10.8%)	
**Any Taxane**			0.056
No	7340 (94.7%)	7285 (94.0%)	
Yes	413 (5.3%)	468 (6.0%)	
**Doxorubicin and BCT (a)**			0.085
No	7707 (99.4%)	7689 (99.2%)	
Yes	46 (0.6%)	64 (0.8%)	
**Doxorubicin and Mastectomy (a)**			0.117
No	7108 (91.7%)	7053 (91.0%)	
Yes	645 (8.3%)	700 (9.0%)	
**Doxorubicin and Radiation (a)**			0.355
No	7314 (94.3%)	7287 (94.0%)	
Yes	439 (5.7%)	466 (6.0%)	
**Doxorubicin and Taxane**			0.838
No	7437 (95.9%)	7442 (96.0%)	
Yes	316 (4.1%)	311 (4.0%)	
**Taxane and BCT (a)**			0.490
No	7718 (99.5%)	7712 (99.5%)	
Yes	35 (0.5%)	41 (0.5%)	
**Taxane and Mastectomy (a)**			0.815
No	7410 (95.6%)	7404 (95.5%)	
Yes	343 (4.4%)	349 (4.5%)	
**Taxane and Radiation (a)**			0.760
No	7477 (96.4%)	7484 (96.5%)	
Yes	276 (3.6%)	269 (3.5%)	
**Radiation and BCT (a)**			0.562
No	7457 (96.2%)	7443 (96.0%)	
Yes	296 (3.8%)	310 (4.0%)	
**Radiation and Mastectomy (a)**			<0.001
No	4447 (57.4%)	5095 (65.7%)	
Yes	3306 (42.6%)	2658 (34.3%)	
**Radiation, Doxorubicin, and Taxane (a)**			0.042
No	7524 (97.0%)	7565 (97.6%)	
Yes	229 (3.0%)	188 (2.4%)	
**BCT, Doxorubicin, Taxane (a)**			0.411
No	7737 (99.8%)	7732 (99.7%)	
Yes	16 (0.2%)	21 (0.3%)	

	White (N = 7753)	Black (N = 7753)	p-value
**BCT, Doxorubicin, Radiation (a)**			0.430
No	7717 (99.5%)	7710 (99.4%)	
Yes	36 (0.5%)	43 (0.6%)	
**BCT, Taxane, Radiation (a)**			0.423
No	7736 (99.8%)	7731 (99.7%)	
Yes	17 (0.2%)	22 (0.3%)	
**Mastectomy, Doxorubicin, Taxane (a)**			0.185
No	7473 (96.4%)	7503 (96.8%)	
Yes	280 (3.6%)	250 (3.2%)	
**Mastectomy, Doxorubicin, Radiation (a)**			0.912
No	7365 (95.0%)	7362 (95.0%)	
Yes	388 (5.0%)	391 (5.0%)	
**Mastectomy, Taxane, Radiation (a)**			0.201
No	7510 (96.9%)	7537 (97.2%)	
Yes	243 (3.1%)	216 (2.8%)	
**Mastectomy, Doxorubicin, Taxane, Radiation (a)**			0.010
No	7545 (97.3%)	7594 (97.9%)	
Yes	208 (2.7%)	159 (2.1%)	
**BCT, Doxorubicin, Taxane, Radiation (a)**			0.841
No	7741 (99.8%)	7740 (99.8%)	
Yes	12 (0.2%)	13 (0.2%)	

Supplemental Table 1b. Controlling for Demographic and Presentation Variables, 1992-2005

	White (N = 7753)	Black (N = 7753)	p-value
**Age**			0.468
Mean (SD)	75.78 (6.99)	75.86 (7.07)	
Median (Q1, Q3)	75.0 (70.0, 80.0)	75.0 (70.0, 80.0)	
**Size (mm)**			0.009
Mean (SD)	26.51 (26.64)	27.67 (26.59)	
Median (Q1, Q3)	20.0 (12.0, 35.0)	20.0 (12.0, 35.0)	
**Number of Positive Nodes**			0.004
Mean (SD)	1.38 (3.69)	1.22 (3.29)	
Median (Q1, Q3)	0.0 (0.0, 1.0)	0.0 (0.0, 1.0)	
**Number of Nodes Examined**			<0.001
Mean (SD)	8.49 (7.90)	8.00 (7.93)	
Median (Q1, Q3)	8.0 (1.0, 14.0)	7.0 (0.0, 14.0)	
**Year of Diagnosis**			0.747
1992	396 (5.1%)	381 (4.9%)	
1993	339 (4.4%)	325 (4.2%)	
1994	280 (3.6%)	322 (4.2%)	
1995	353 (4.6%)	327 (4.2%)	
1996	318 (4.1%)	338 (4.4%)	

	White (N = 7753)	Black (N = 7753)	p-value
1997	320 (4.1%)	328 (4.2%)	
1998	376 (4.8%)	329 (4.2%)	
1999	287 (3.7%)	302 (3.9%)	
2000	849 (11.0%)	866 (11.2%)	
2001	809 (10.4%)	825 (10.6%)	
2002	880 (11.4%)	874 (11.3%)	
2003	881 (11.4%)	863 (11.1%)	
2004	824 (10.6%)	835 (10.8%)	
2005	841 (10.8%)	838 (10.8%)	
**Registry**			0.950
Connecticut	309 (4.0%)	326 (4.2%)	
Detroit	1881 (24.3%)	1842 (23.8%)	
Hawaii	* (*)	* (*)	
Iowa	69 (0.9%)	69 (0.9%)	
New Mexico	* (*)	* (*)	
Seattle	104 (1.3%)	104 (1.3%)	
Utah	* (*)	* (*)	
Kentucky	210 (2.7%)	238 (3.1%)	
Louisiana	865 (11.2%)	908 (11.7%)	
New Jersey	819 (10.6%)	800 (10.3%)	
Georgia	1795 (23.2%)	1801 (23.2%)	
California	1658 (21.4%)	1625 (21.0%)	
**AJCC Stage**			0.993
Stage I	3015 (38.9%)	3015 (38.9%)	
Stage II	3098 (40.0%)	3085 (39.8%)	
Stage III	887 (11.4%)	897 (11.6%)	
Stage IV	753 (9.7%)	756 (9.8%)	
**Tumor Grade**			0.258
Grade I	1051 (13.6%)	1037 (13.4%)	
Grade II	2435 (31.4%)	2419 (31.2%)	
Grade III	2584 (33.3%)	2645 (34.1%)	
Grade IV	114 (1.5%)	143 (1.8%)	
Missing	1569 (20.2%)	1509 (19.5%)	
**ER Status**			0.900
Negative	1554 (20.0%)	1577 (20.3%)	
Positive	4331 (55.9%)	4315 (55.7%)	
Missing	1868 (24.1%)	1861 (24.0%)	
**PR Status**			0.946
Negative	2431 (31.4%)	2412 (31.1%)	
Positive	3396 (43.8%)	3406 (43.9%)	
Missing	1926 (24.8%)	1935 (25.0%)	
**Tumor Size**			0.958
0-.9cm	1126 (14.5%)	1105 (14.3%)	
1-1.9	2160 (27.9%)	2149 (27.7%)	
2-2.9	1584 (20.4%)	1572 (20.3%)	

	White (N = 7753)	Black (N = 7753)	p-value
3-3.9	864 (11.1%)	855 (11.0%)	
4+cm	1509 (19.5%)	1555 (20.1%)	
Missing	510 (6.6%)	517 (6.7%)	
**Congestive Heart Failure**			0.463
No	6171 (79.6%)	6134 (79.1%)	
Yes	1582 (20.4%)	1619 (20.9%)	
**Past Arrhythmia**			0.381
No	6743 (87.0%)	6706 (86.5%)	
Yes	1010 (13.0%)	1047 (13.5%)	
**Past Myocardial Infarction**			0.802
No	7452 (96.1%)	7458 (96.2%)	
Yes	301 (3.9%)	295 (3.8%)	
**Angina**			0.499
No	7277 (93.9%)	7297 (94.1%)	
Yes	476 (6.1%)	456 (5.9%)	
**Valvular Heart Disease**			0.154
No	7104 (91.6%)	7054 (91.0%)	
Yes	649 (8.4%)	699 (9.0%)	
**Hypertension (uncomplicated)**			0.909
No	3251 (41.9%)	3258 (42.0%)	
Yes	4502 (58.1%)	4495 (58.0%)	
**Hypertension (complications)**			0.743
No	5726 (73.9%)	5708 (73.6%)	
Yes	2027 (26.1%)	2045 (26.4%)	
**Diabetes (uncomplicated)**			0.667
No	5875 (75.8%)	5852 (75.5%)	
Yes	1878 (24.2%)	1901 (24.5%)	
**Diabetes (complications)**			0.923
No	6771 (87.3%)	6767 (87.3%)	
Yes	982 (12.7%)	986 (12.7%)	
**Kidney Disease**			0.316
None	7094 (91.5%)	7065 (91.1%)	
Renal Dysfunction	164 (2.1%)	151 (1.9%)	
Renal Failure	495 (6.4%)	537 (6.9%)	
**Liver disease**			0.874
No	7674 (99.0%)	7672 (99.0%)	
Yes	79 (1.0%)	81 (1.0%)	
**Chronic Lung Disease**			0.884
No	6346 (81.9%)	6339 (81.8%)	
Yes	1407 (18.1%)	1414 (18.2%)	
**Dementia**			0.810
No	7151 (92.2%)	7159 (92.3%)	
Yes	602 (7.8%)	594 (7.7%)	
**Ischemic event**			0.253
No	7514 (96.9%)	7538 (97.2%)	

	White (N = 7753)	Black (N = 7753)	p-value
Yes	239 (3.1%)	215 (2.8%)	
**Stroke**			0.693
No	7488 (96.6%)	7479 (96.5%)	
Yes	265 (3.4%)	274 (3.5%)	
**Hypothyroidism**			0.492
No	6869 (88.6%)	6896 (88.9%)	
Yes	884 (11.4%)	857 (11.1%)	
**Paralysis**			0.492
No	7360 (94.9%)	7341 (94.7%)	
Yes	393 (5.1%)	412 (5.3%)	
**Collagen Vascular Disease**			0.940
No	7383 (95.2%)	7385 (95.3%)	
Yes	370 (4.8%)	368 (4.7%)	
**Coagulopathy**			0.512
No	7410 (95.6%)	7393 (95.4%)	
Yes	343 (4.4%)	360 (4.6%)	
**Chronic Obstructive Pulmonary Disease**			0.805
No	6301 (81.3%)	6289 (81.1%)	
Yes	1452 (18.7%)	1464 (18.9%)	
**Peptic Ulcer Disease**			0.262
No	7737 (99.8%)	7730 (99.7%)	
Yes	16 (0.2%)	23 (0.3%)	
**Depression**			0.915
No	7339 (94.7%)	7336 (94.6%)	
Yes	414 (5.3%)	417 (5.4%)	
**Cushing's Disease**			0.705
No	* (100%)	* (100%)	
Yes	* (0%)	* (0%)	
**Graves Disease**			0.414
No	* (100%)	* (100%)	
Yes	* (0%)	* (0%)	
**Post-Inflammatory Pulmonary Fibrosis**			0.406
No	7675 (99.0%)	7685 (99.1%)	
Yes	78 (1.0%)	68 (0.9%)	
**Any Breast Conserving Therapy**			0.019
No BCT	6199 (80.0%)	6080 (78.4%)	
BCT	1554 (20.0%)	1673 (21.6%)	
**Any Mastectomy**			<0.001
No Mastectomy	2293 (29.6%)	2684 (34.6%)	
Mastectomy	5460 (70.4%)	5069 (65.4%)	
**Any Radiation**			<0.001
No	4465 (57.6%)	4711 (60.8%)	
Yes	3288 (42.4%)	3042 (39.2%)	
**Any Chemotherapy**			0.115
No	5709 (73.6%)	5795 (74.7%)	

	White (N = 7753)	Black (N = 7753)	p-value
Yes	2044 (26.4%)	1958 (25.3%)	
**Any Doxorubicin**			0.004
No	6802 (87.7%)	6917 (89.2%)	
Yes	951 (12.3%)	836 (10.8%)	
**Any Taxane**			<0.001
No	7172 (92.5%)	7285 (94.0%)	
Yes	581 (7.5%)	468 (6.0%)	
**Doxorubicin and BCT**			0.036
No	7557 (97.5%)	7596 (98.0%)	
Yes	196 (2.5%)	157 (2.0%)	
**Doxorubicin and Mastectomy**			0.003
No	7069 (91.2%)	7171 (92.5%)	
Yes	684 (8.8%)	582 (7.5%)	
**Doxorubicin and Radiation**			<0.001
No	7183 (92.6%)	7298 (94.1%)	
Yes	570 (7.4%)	455 (5.9%)	
**Doxorubicin and Taxane**			<0.001
No	7351 (94.8%)	7442 (96.0%)	
Yes	402 (5.2%)	311 (4.0%)	
**Taxane and BCT**			0.007
No	7622 (98.3%)	7662 (98.8%)	
Yes	131 (1.7%)	91 (1.2%)	
**Taxane and Mastectomy**			0.001
No	7392 (95.3%)	7472 (96.4%)	
Yes	361 (4.7%)	281 (3.6%)	
**Taxane and Radiation**			<0.001
No	7388 (95.3%)	7490 (96.6%)	
Yes	365 (4.7%)	263 (3.4%)	
**Radiation and BCT**			0.871
No	6678 (86.1%)	6671 (86.0%)	
Yes	1075 (13.9%)	1082 (14.0%)	
**Radiation and Mastectomy**			<0.001
No	5748 (74.1%)	6024 (77.7%)	
Yes	2005 (25.9%)	1729 (22.3%)	
**Radiation, Doxorubicin, Taxane**			<0.001
No	7471 (96.4%)	7569 (97.6%)	
Yes	282 (3.6%)	184 (2.4%)	
**BCT, Doxorubicin, Taxane**			0.268
No	7677 (99.0%)	7690 (99.2%)	
Yes	76 (1.0%)	63 (0.8%)	
**BCT, Doxorubicin, Radiation**			0.017
No	7591 (97.9%)	7631 (98.4%)	
Yes	162 (2.1%)	122 (1.6%)	
**BCT, Taxane, Radiation**			0.012
No	7655 (98.7%)	7687 (99.1%)	

	White (N = 7753)	Black (N = 7753)	p-value
Yes	98 (1.3%)	66 (0.9%)	
**Mastectomy, Doxorubicin, Taxane**			<0.001
No	7469 (96.3%)	7554 (97.4%)	
Yes	284 (3.7%)	199 (2.6%)	
**Mastectomy, Doxorubicin, Radiation**			<0.001
No	7379 (95.2%)	7467 (96.3%)	
Yes	374 (4.8%)	286 (3.7%)	
**Mastectomy, Taxane, Radiation**			<0.001
No	7524 (97.0%)	7594 (97.9%)	
Yes	229 (3.0%)	159 (2.1%)	
**Mastectomy, Doxorubicin, Taxane, Radiation**			<0.001
No	7557 (97.5%)	7638 (98.5%)	
Yes	196 (2.5%)	115 (1.5%)	
**BCT, Doxorubicin, Taxane, Radiation**			0.112
No	7687 (99.1%)	7704 (99.4%)	
Yes	66 (0.9%)	49 (0.6%)	
**Any Breast Conserving Therapy (a)**			0.001
No BCT	7249 (93.5%)	7145 (92.2%)	
BCT	504 (6.5%)	608 (7.8%)	
**Any Mastectomy (a)**			<0.001
No Mastectomy	1137 (14.7%)	1477 (19.1%)	
Mastectomy	6616 (85.3%)	6276 (80.9%)	
**Any Radiation (a)**			<0.001
No	4356 (56.2%)	4604 (59.4%)	
Yes	3397 (43.8%)	3149 (40.6%)	
**Any Chemotherapy (a)**			0.011
No	5577 (71.9%)	5717 (73.7%)	
Yes	2176 (28.1%)	2036 (26.3%)	
**Any Doxorubicin**			0.004
No	6802 (87.7%)	6917 (89.2%)	
Yes	951 (12.3%)	836 (10.8%)	
**Any Taxane**			<0.001
No	7172 (92.5%)	7285 (94.0%)	
Yes	581 (7.5%)	468 (6.0%)	
**Doxorubicin and BCT (a)**			0.491
No	7681 (99.1%)	7689 (99.2%)	
Yes	72 (0.9%)	64 (0.8%)	
**Doxorubicin and Mastectomy (a)**			0.001
No	6929 (89.4%)	7053 (91.0%)	
Yes	824 (10.6%)	700 (9.0%)	
**Doxorubicin and Radiation (a)**			<0.001
No	7165 (92.4%)	7287 (94.0%)	
Yes	588 (7.6%)	466 (6.0%)	
**Doxorubicin and Taxane**			<0.001

	White (N = 7753)	Black (N = 7753)	p-value
No	7351 (94.8%)	7442 (96.0%)	
Yes	402 (5.2%)	311 (4.0%)	
**Taxane and BCT (a)**			0.047
No	7692 (99.2%)	7712 (99.5%)	
Yes	61 (0.8%)	41 (0.5%)	
**Taxane and Mastectomy (a)**			<0.001
No	7299 (94.1%)	7404 (95.5%)	
Yes	454 (5.9%)	349 (4.5%)	
**Taxane and Radiation (a)**			<0.001
No	7382 (95.2%)	7484 (96.5%)	
Yes	371 (4.8%)	269 (3.5%)	
**Radiation and BCT (a)**			0.454
No	7461 (96.2%)	7443 (96.0%)	
Yes	292 (3.8%)	310 (4.0%)	
**Radiation and Mastectomy (a)**			<0.001
No	4829 (62.3%)	5095 (65.7%)	
Yes	2924 (37.7%)	2658 (34.3%)	
**Radiation, Doxorubicin, and Taxane (a)**			<0.001
No	7467 (96.3%)	7565 (97.6%)	
Yes	286 (3.7%)	188 (2.4%)	
**BCT, Doxorubicin, Taxane (a)**			0.317
No	7725 (99.6%)	7732 (99.7%)	
Yes	28 (0.4%)	21 (0.3%)	
**BCT, Doxorubicin, Radiation (a)**			0.224
No	7698 (99.3%)	7710 (99.4%)	
Yes	55 (0.7%)	43 (0.6%)	
**BCT, Taxane, Radiation (a)**			0.066
No	7717 (99.5%)	7731 (99.7%)	
Yes	36 (0.5%)	22 (0.3%)	
**Mastectomy, Doxorubicin, Taxane (a)**			<0.001
No	7410 (95.6%)	7503 (96.8%)	
Yes	343 (4.4%)	250 (3.2%)	
**Mastectomy, Doxorubicin, Radiation (a)**			<0.001
No	7248 (93.5%)	7362 (95.0%)	
Yes	505 (6.5%)	391 (5.0%)	
**Mastectomy, Taxane, Radiation (a)**			<0.001
No	7448 (96.1%)	7537 (97.2%)	
Yes	305 (3.9%)	216 (2.8%)	
**Mastectomy, Doxorubicin, Taxane, Radiation (a)**			<0.001
No	7505 (96.8%)	7594 (97.9%)	
Yes	248 (3.2%)	159 (2.1%)	
**BCT, Doxorubicin, Taxane, Radiation (a)**			0.170
No	7732 (99.7%)	7740 (99.8%)	

	White (N = 7753)	Black (N = 7753)	p-value
Yes	21 (0.3%)	13 (0.2%)	

Supplemental Table 1c. Controlling for Demographic, Presentation, and Treatment Variables, 1992-2005

	White (N = 7753)	Black (N = 7753)	p-value
**Age**			0.616
Mean (SD)	75.92 (7.07)	75.86 (7.07)	
Median (Q1, Q3)	75.0 (70.0, 81.0)	75.0 (70.0, 80.0)	
**Size (mm)**			0.021
Mean (SD)	26.61 (28.88)	27.67 (26.59)	
Median (Q1, Q3)	20.0 (12.0, 35.0)	20.0 (12.0, 35.0)	
**Number of Positive Nodes**			0.437
Mean (SD)	1.18 (3.20)	1.22 (3.29)	
Median (Q1, Q3)	0.0 (0.0, 1.0)	0.0 (0.0, 1.0)	
**Number of Nodes Examined**			0.981
Mean (SD)	8.01 (7.88)	8.00 (7.93)	
Median (Q1, Q3)	7.0 (0.0, 14.0)	7.0 (0.0, 14.0)	
**Year of Diagnosis**			0.971
1992	385 (5.0%)	381 (4.9%)	
1993	320 (4.1%)	325 (4.2%)	
1994	333 (4.3%)	322 (4.2%)	
1995	305 (3.9%)	327 (4.2%)	
1996	335 (4.3%)	338 (4.4%)	
1997	359 (4.6%)	328 (4.2%)	
1998	349 (4.5%)	329 (4.2%)	
1999	282 (3.6%)	302 (3.9%)	
2000	869 (11.2%)	866 (11.2%)	
2001	780 (10.1%)	825 (10.6%)	
2002	886 (11.4%)	874 (11.3%)	
2003	862 (11.1%)	863 (11.1%)	
2004	834 (10.8%)	835 (10.8%)	
2005	854 (11.0%)	838 (10.8%)	
**Registry**			0.982
Connecticut	325 (4.2%)	326 (4.2%)	
Detroit	1774 (22.9%)	1842 (23.8%)	
Hawaii	* (*)	* (*)	
Iowa	78 (1.0%)	69 (0.9%)	
New Mexico	* (*)	* (*)	
Seattle	108 (1.4%)	104 (1.3%)	
Utah	* (*)	* (*)	
Kentucky	236 (3.0%)	238 (3.1%)	
Louisiana	901 (11.6%)	908 (11.7%)	
New Jersey	804 (10.4%)	800 (10.3%)	
Georgia	1814 (23.4%)	1801 (23.2%)	
California	1670 (21.5%)	1625 (21.0%)	

	White (N = 7753)	Black (N = 7753)	p-value
**AJCC Stage**			0.906
Stage I	3050 (39.3%)	3015 (38.9%)	
Stage II	3066 (39.5%)	3085 (39.8%)	
Stage III	875 (11.3%)	897 (11.6%)	
Stage IV	762 (9.8%)	756 (9.8%)	
**Tumor Grade**			0.925
Grade I	1053 (13.6%)	1037 (13.4%)	
Grade II	2397 (30.9%)	2419 (31.2%)	
Grade III	2614 (33.7%)	2645 (34.1%)	
Grade IV	151 (1.9%)	143 (1.8%)	
Missing	1538 (19.8%)	1509 (19.5%)	
**ER Status**			0.898
Negative	1598 (20.6%)	1577 (20.3%)	
Positive	4290 (55.3%)	4315 (55.7%)	
Missing	1865 (24.1%)	1861 (24.0%)	
**PR Status**			0.776
Negative	2453 (31.6%)	2412 (31.1%)	
Positive	3377 (43.6%)	3406 (43.9%)	
Missing	1923 (24.8%)	1935 (25.0%)	
**Tumor Size**			0.988
0-.9cm	1100 (14.2%)	1105 (14.3%)	
1-1.9	2188 (28.2%)	2149 (27.7%)	
2-2.9	1573 (20.3%)	1572 (20.3%)	
3-3.9	851 (11.0%)	855 (11.0%)	
4+cm	1530 (19.7%)	1555 (20.1%)	
Missing	511 (6.6%)	517 (6.7%)	
**Congestive Heart Failure**			0.427
No	6174 (79.6%)	6134 (79.1%)	
Yes	1579 (20.4%)	1619 (20.9%)	
**Past Arrhythmia**			0.200
No	6760 (87.2%)	6706 (86.5%)	
Yes	993 (12.8%)	1047 (13.5%)	
**Past Myocardial Infarction**			0.611
No	7470 (96.3%)	7458 (96.2%)	
Yes	283 (3.7%)	295 (3.8%)	
**Angina**			0.758
No	7306 (94.2%)	7297 (94.1%)	
Yes	447 (5.8%)	456 (5.9%)	
**Valvular Heart Disease**			0.652
No	7070 (91.2%)	7054 (91.0%)	
Yes	683 (8.8%)	699 (9.0%)	
**Hypertension (uncomplicated)**			0.474
No	3214 (41.5%)	3258 (42.0%)	
Yes	4539 (58.5%)	4495 (58.0%)	
**Hypertension (complications)**			0.323

	White (N = 7753)	Black (N = 7753)	p-value
No	5762 (74.3%)	5708 (73.6%)	
Yes	1991 (25.7%)	2045 (26.4%)	
**Diabetes (uncomplicated)**			0.615
No	5825 (75.1%)	5852 (75.5%)	
Yes	1928 (24.9%)	1901 (24.5%)	
**Diabetes (complications)**			0.513
No	6794 (87.6%)	6767 (87.3%)	
Yes	959 (12.4%)	986 (12.7%)	
**Kidney Disease**			0.473
None	7103 (91.6%)	7065 (91.1%)	
Renal Dysfunction	151 (1.9%)	151 (1.9%)	
Renal Failure	499 (6.4%)	537 (6.9%)	
**Liver disease**			0.629
No	7678 (99.0%)	7672 (99.0%)	
Yes	75 (1.0%)	81 (1.0%)	
**Chronic Lung Disease**			0.787
No	6352 (81.9%)	6339 (81.8%)	
Yes	1401 (18.1%)	1414 (18.2%)	
**Dementia**			0.716
No	7171 (92.5%)	7159 (92.3%)	
Yes	582 (7.5%)	594 (7.7%)	
**Ischemic event**			0.342
No	7557 (97.5%)	7538 (97.2%)	
Yes	196 (2.5%)	215 (2.8%)	
**Stroke**			0.598
No	7491 (96.6%)	7479 (96.5%)	
Yes	262 (3.4%)	274 (3.5%)	
**Hypothyroidism**			0.424
No	6927 (89.3%)	6896 (88.9%)	
Yes	826 (10.7%)	857 (11.1%)	
**Paralysis**			0.274
No	7371 (95.1%)	7341 (94.7%)	
Yes	382 (4.9%)	412 (5.3%)	
**Collagen Vascular Disease**			0.704
No	7395 (95.4%)	7385 (95.3%)	
Yes	358 (4.6%)	368 (4.7%)	
**Coagulopathy**			0.970
No	7392 (95.3%)	7393 (95.4%)	
Yes	361 (4.7%)	360 (4.6%)	
**Chronic Obstructive Pulmonary Disease**			0.742
No	6305 (81.3%)	6289 (81.1%)	
Yes	1448 (18.7%)	1464 (18.9%)	
**Peptic Ulcer Disease**			0.275
No	7722 (99.6%)	7730 (99.7%)	
Yes	31 (0.4%)	23 (0.3%)	

	White (N = 7753)	Black (N = 7753)	p-value
**Depression**			0.329
No	7363 (95.0%)	7336 (94.6%)	
Yes	390 (5.0%)	417 (5.4%)	
**Cushing's Disease**			0.705
No	* (100%)	* (100%)	
Yes	* (0%)	* (0%)	
**Graves Disease**			0.564
No	* (100%)	* (100%)	
Yes	* (0%)	* (0%)	
**Post-Inflammatory Pulmonary Fibrosis**			0.597
No	7691 (99.2%)	7685 (99.1%)	
Yes	62 (0.8%)	68 (0.9%)	
**Any Breast Conserving Therapy**			0.484
No BCT	6044 (78.0%)	6080 (78.4%)	
BCT	1709 (22.0%)	1673 (21.6%)	
**Any Mastectomy**			0.637
No Mastectomy	2712 (35.0%)	2684 (34.6%)	
Mastectomy	5041 (65.0%)	5069 (65.4%)	
**Any Radiation**			0.755
No	4692 (60.5%)	4711 (60.8%)	
Yes	3061 (39.5%)	3042 (39.2%)	
**Any Chemotherapy**			0.450
No	5754 (74.2%)	5795 (74.7%)	
Yes	1999 (25.8%)	1958 (25.3%)	
**Any Doxorubicin**			0.520
No	6892 (88.9%)	6917 (89.2%)	
Yes	861 (11.1%)	836 (10.8%)	
**Any Taxane**			0.518
No	7304 (94.2%)	7285 (94.0%)	
Yes	449 (5.8%)	468 (6.0%)	
**Doxorubicin and BCT**			0.865
No	7593 (97.9%)	7596 (98.0%)	
Yes	160 (2.1%)	157 (2.0%)	
**Doxorubicin and Mastectomy**			0.351
No	7140 (92.1%)	7171 (92.5%)	
Yes	613 (7.9%)	582 (7.5%)	
**Doxorubicin and Radiation**			0.542
No	7280 (93.9%)	7298 (94.1%)	
Yes	473 (6.1%)	455 (5.9%)	
**Doxorubicin and Taxane**			0.903
No	7439 (95.9%)	7442 (96.0%)	
Yes	314 (4.1%)	311 (4.0%)	
**Taxane and BCT**			0.940
No	7663 (98.8%)	7662 (98.8%)	
Yes	90 (1.2%)	91 (1.2%)	

	White (N = 7753)	Black (N = 7753)	p-value
**Taxane and Mastectomy**			0.897
No	7475 (96.4%)	7472 (96.4%)	
Yes	278 (3.6%)	281 (3.6%)	
**Taxane and Radiation**			0.755
No	7497 (96.7%)	7490 (96.6%)	
Yes	256 (3.3%)	263 (3.4%)	
**Radiation and BCT**			0.628
No	6650 (85.8%)	6671 (86.0%)	
Yes	1103 (14.2%)	1082 (14.0%)	
**Radiation and Mastectomy**			0.877
No	6016 (77.6%)	6024 (77.7%)	
Yes	1737 (22.4%)	1729 (22.3%)	
**Radiation, Doxorubicin, Taxane**			0.753
No	7563 (97.5%)	7569 (97.6%)	
Yes	190 (2.5%)	184 (2.4%)	
**BCT, Doxorubicin, Taxane**			0.716
No	7694 (99.2%)	7690 (99.2%)	
Yes	59 (0.8%)	63 (0.8%)	
**BCT, Doxorubicin, Radiation**			0.656
No	7624 (98.3%)	7631 (98.4%)	
Yes	129 (1.7%)	122 (1.6%)	
**BCT, Taxane, Radiation**			0.930
No	7688 (99.2%)	7687 (99.1%)	
Yes	65 (0.8%)	66 (0.9%)	
**Mastectomy, Doxorubicin, Taxane**			0.485
No	7540 (97.3%)	7554 (97.4%)	
Yes	213 (2.7%)	199 (2.6%)	
**Mastectomy, Doxorubicin, Radiation**			0.528
No	7452 (96.1%)	7467 (96.3%)	
Yes	301 (3.9%)	286 (3.7%)	
**Mastectomy, Taxane, Radiation**			0.866
No	7591 (97.9%)	7594 (97.9%)	
Yes	162 (2.1%)	159 (2.1%)	
**Mastectomy, Doxorubicin, Taxane, Radiation**			0.366
No	7624 (98.3%)	7638 (98.5%)	
Yes	129 (1.7%)	115 (1.5%)	
**BCT, Doxorubicin, Taxane, Radiation**			0.603
No	7709 (99.4%)	7704 (99.4%)	
Yes	44 (0.6%)	49 (0.6%)	
**Any Breast Conserving Therapy (a)**			0.015
No BCT	7224 (93.2%)	7145 (92.2%)	
BCT	529 (6.8%)	608 (7.8%)	
**Any Mastectomy (a)**			0.072
No Mastectomy	1390 (17.9%)	1477 (19.1%)	

	White (N = 7753)	Black (N = 7753)	p-value
Mastectomy	6363 (82.1%)	6276 (80.9%)	
**Any Radiation (a)**			0.683
No	4579 (59.1%)	4604 (59.4%)	
Yes	3174 (40.9%)	3149 (40.6%)	
**Any Chemotherapy (a)**			0.076
No	5619 (72.5%)	5717 (73.7%)	
Yes	2134 (27.5%)	2036 (26.3%)	
**Any Doxorubicin**			0.520
No	6892 (88.9%)	6917 (89.2%)	
Yes	861 (11.1%)	836 (10.8%)	
**Any Taxane**			0.518
No	7304 (94.2%)	7285 (94.0%)	
Yes	449 (5.8%)	468 (6.0%)	
**Doxorubicin and BCT (a)**			1.000
No	7689 (99.2%)	7689 (99.2%)	
Yes	64 (0.8%)	64 (0.8%)	
**Doxorubicin and Mastectomy (a)**			0.405
No	7023 (90.6%)	7053 (91.0%)	
Yes	730 (9.4%)	700 (9.0%)	
**Doxorubicin and Radiation (a)**			0.525
No	7268 (93.7%)	7287 (94.0%)	
Yes	485 (6.3%)	466 (6.0%)	
**Doxorubicin and Taxane**			0.903
No	7439 (95.9%)	7442 (96.0%)	
Yes	314 (4.1%)	311 (4.0%)	
**Taxane and BCT (a)**			0.214
No	7700 (99.3%)	7712 (99.5%)	
Yes	53 (0.7%)	41 (0.5%)	
**Taxane and Mastectomy (a)**			0.611
No	7417 (95.7%)	7404 (95.5%)	
Yes	336 (4.3%)	349 (4.5%)	
**Taxane and Radiation (a)**			0.791
No	7490 (96.6%)	7484 (96.5%)	
Yes	263 (3.4%)	269 (3.5%)	
**Radiation and BCT (a)**			0.241
No	7471 (96.4%)	7443 (96.0%)	
Yes	282 (3.6%)	310 (4.0%)	
**Radiation and Mastectomy (a)**			0.457
No	5051 (65.1%)	5095 (65.7%)	
Yes	2702 (34.9%)	2658 (34.3%)	
**Radiation, Doxorubicin, and Taxane (a)**			0.756
No	7559 (97.5%)	7565 (97.6%)	
Yes	194 (2.5%)	188 (2.4%)	
**BCT, Doxorubicin, Taxane (a)**			0.079
No	7719 (99.6%)	7732 (99.7%)	

	White (N = 7753)	Black (N = 7753)	p-value
Yes	34 (0.4%)	21 (0.3%)	
**BCT, Doxorubicin, Radiation (a)**			0.578
No	7715 (99.5%)	7710 (99.4%)	
Yes	38 (0.5%)	43 (0.6%)	
**BCT, Taxane, Radiation (a)**			0.563
No	7727 (99.7%)	7731 (99.7%)	
Yes	26 (0.3%)	22 (0.3%)	
**Mastectomy, Doxorubicin, Taxane (a)**			0.964
No	7502 (96.8%)	7503 (96.8%)	
Yes	251 (3.2%)	250 (3.2%)	
**Mastectomy, Doxorubicin, Radiation (a)**			0.468
No	7342 (94.7%)	7362 (95.0%)	
Yes	411 (5.3%)	391 (5.0%)	
**Mastectomy, Taxane, Radiation (a)**			0.883
No	7540 (97.3%)	7537 (97.2%)	
Yes	213 (2.7%)	216 (2.8%)	
**Mastectomy, Doxorubicin, Taxane, Radiation (a)**			0.866
No	7591 (97.9%)	7594 (97.9%)	
Yes	162 (2.1%)	159 (2.1%)	
**BCT, Doxorubicin, Taxane, Radiation (a)**			0.369
No	7735 (99.8%)	7740 (99.8%)	
Yes	18 (0.2%)	13 (0.2%)	

Supplemental Table 1d. Controlling for Demographic, Presentation, and Augmented Treatment Variables (a = Augmented Definitions), 1992-2005

	White (N = 7753)	Black (N = 7753)	p-value
**Age**			0.804
Mean (SD)	75.89 (7.12)	75.86 (7.07)	
Median (Q1, Q3)	75.0 (70.0, 81.0)	75.0 (70.0, 80.0)	
**Size (mm)**			0.048
Mean (SD)	26.85 (23.32)	27.67 (26.59)	
Median (Q1, Q3)	20.0 (12.0, 35.0)	20.0 (12.0, 35.0)	
**Number of Positive Nodes**			0.668
Mean (SD)	1.24 (3.35)	1.22 (3.29)	
Median (Q1, Q3)	0.0 (0.0, 1.0)	0.0 (0.0, 1.0)	
**Number of Nodes Examined**			0.728
Mean (SD)	8.05 (7.98)	8.00 (7.93)	
Median (Q1, Q3)	7.0 (0.0, 14.0)	7.0 (0.0, 14.0)	
**Year of Diagnosis**			0.949
1992	403 (5.2%)	381 (4.9%)	
1993	346 (4.5%)	325 (4.2%)	
1994	341 (4.4%)	322 (4.2%)	
1995	311 (4.0%)	327 (4.2%)	

	White (N = 7753)	Black (N = 7753)	p-value
1996	361 (4.7%)	338 (4.4%)	
1997	308 (4.0%)	328 (4.2%)	
1998	327 (4.2%)	329 (4.2%)	
1999	316 (4.1%)	302 (3.9%)	
2000	902 (11.6%)	866 (11.2%)	
2001	796 (10.3%)	825 (10.6%)	
2002	858 (11.1%)	874 (11.3%)	
2003	839 (10.8%)	863 (11.1%)	
2004	825 (10.6%)	835 (10.8%)	
2005	820 (10.6%)	838 (10.8%)	
**Registry**			0.962
Connecticut	333 (4.3%)	326 (4.2%)	
Detroit	1822 (23.5%)	1842 (23.8%)	
Hawaii	* (*)	* (*)	
Iowa	67 (0.9%)	69 (0.9%)	
New Mexico	* (*)	* (*)	
Seattle	96 (1.2%)	104 (1.3%)	
Utah	* (*)	* (*)	
Kentucky	229 (3.0%)	238 (3.1%)	
Louisiana	867 (11.2%)	908 (11.7%)	
New Jersey	822 (10.6%)	800 (10.3%)	
Georgia	1833 (23.6%)	1801 (23.2%)	
California	1635 (21.1%)	1625 (21.0%)	
**AJCC Stage**			0.972
Stage I	3012 (38.8%)	3015 (38.9%)	
Stage II	3067 (39.6%)	3085 (39.8%)	
Stage III	902 (11.6%)	897 (11.6%)	
Stage IV	772 (10.0%)	756 (9.8%)	
**Tumor Grade**			0.708
Grade I	997 (12.9%)	1037 (13.4%)	
Grade II	2376 (30.6%)	2419 (31.2%)	
Grade III	2695 (34.8%)	2645 (34.1%)	
Grade IV	153 (2.0%)	143 (1.8%)	
Missing	1532 (19.8%)	1509 (19.5%)	
**ER Status**			0.893
Negative	1599 (20.6%)	1577 (20.3%)	
Positive	4309 (55.6%)	4315 (55.7%)	
Missing	1845 (23.8%)	1861 (24.0%)	
**PR Status**			0.849
Negative	2445 (31.5%)	2412 (31.1%)	
Positive	3386 (43.7%)	3406 (43.9%)	
Missing	1922 (24.8%)	1935 (25.0%)	
**Tumor Size**			0.979
0-.9cm	1109 (14.3%)	1105 (14.3%)	
1-1.9	2103 (27.1%)	2149 (27.7%)	

	White (N = 7753)	Black (N = 7753)	p-value
2-2.9	1592 (20.5%)	1572 (20.3%)	
3-3.9	854 (11.0%)	855 (11.0%)	
4+cm	1574 (20.3%)	1555 (20.1%)	
Missing	521 (6.7%)	517 (6.7%)	
**Congestive Heart Failure**			0.665
No	6112 (78.8%)	6134 (79.1%)	
Yes	1641 (21.2%)	1619 (20.9%)	
**Past Arrhythmia**			0.200
No	6760 (87.2%)	6706 (86.5%)	
Yes	993 (12.8%)	1047 (13.5%)	
**Past Myocardial Infarction**			0.641
No	7469 (96.3%)	7458 (96.2%)	
Yes	284 (3.7%)	295 (3.8%)	
**Angina**			0.864
No	7302 (94.2%)	7297 (94.1%)	
Yes	451 (5.8%)	456 (5.9%)	
**Valvular Heart Disease**			0.673
No	7069 (91.2%)	7054 (91.0%)	
Yes	684 (8.8%)	699 (9.0%)	
**Hypertension (uncomplicated)**			0.974
No	3256 (42.0%)	3258 (42.0%)	
Yes	4497 (58.0%)	4495 (58.0%)	
**Hypertension (complications)**			0.547
No	5741 (74.0%)	5708 (73.6%)	
Yes	2012 (26.0%)	2045 (26.4%)	
**Diabetes (uncomplicated)**			0.627
No	5878 (75.8%)	5852 (75.5%)	
Yes	1875 (24.2%)	1901 (24.5%)	
**Diabetes (complications)**			0.497
No	6795 (87.6%)	6767 (87.3%)	
Yes	958 (12.4%)	986 (12.7%)	
**Kidney Disease**			0.243
None	7116 (91.8%)	7065 (91.1%)	
Renal Dysfunction	152 (2.0%)	151 (1.9%)	
Renal Failure	485 (6.3%)	537 (6.9%)	
**Liver disease**			0.811
No	7675 (99.0%)	7672 (99.0%)	
Yes	78 (1.0%)	81 (1.0%)	
**Chronic Lung Disease**			0.292
No	6288 (81.1%)	6339 (81.8%)	
Yes	1465 (18.9%)	1414 (18.2%)	
**Dementia**			0.762
No	7169 (92.5%)	7159 (92.3%)	
Yes	584 (7.5%)	594 (7.7%)	
**Ischemic event**			0.884

	White (N = 7753)	Black (N = 7753)	p-value
No	7535 (97.2%)	7538 (97.2%)	
Yes	218 (2.8%)	215 (2.8%)	
**Stroke**			0.096
No	7516 (96.9%)	7479 (96.5%)	
Yes	237 (3.1%)	274 (3.5%)	
**Hypothyroidism**			0.857
No	6903 (89.0%)	6896 (88.9%)	
Yes	850 (11.0%)	857 (11.1%)	
**Paralysis**			0.384
No	7365 (95.0%)	7341 (94.7%)	
Yes	388 (5.0%)	412 (5.3%)	
**Collagen Vascular Disease**			0.940
No	7383 (95.2%)	7385 (95.3%)	
Yes	370 (4.8%)	368 (4.7%)	
**Coagulopathy**			0.597
No	7379 (95.2%)	7393 (95.4%)	
Yes	374 (4.8%)	360 (4.6%)	
**Chronic Obstructive Pulmonary Disease**			0.262
No	6234 (80.4%)	6289 (81.1%)	
Yes	1519 (19.6%)	1464 (18.9%)	
**Peptic Ulcer Disease**			0.647
No	7733 (99.7%)	7730 (99.7%)	
Yes	20 (0.3%)	23 (0.3%)	
**Depression**			0.972
No	7335 (94.6%)	7336 (94.6%)	
Yes	418 (5.4%)	417 (5.4%)	
**Cushing's Disease**			0.317
No	7752 (100.0%)	7750 (100.0%)	
Yes	1 (0.0%)	3 (0.0%)	
**Graves Disease**			0.157
No	7753 (100.0%)	7751 (100.0%)	
Yes	0 (0.0%)	2 (0.0%)	
**Post-Inflammatory Pulmonary Fibrosis**			0.794
No	7688 (99.2%)	7685 (99.1%)	
Yes	65 (0.8%)	68 (0.9%)	
**Any Breast Conserving Therapy**			0.326
No BCT	6130 (79.1%)	6080 (78.4%)	
BCT	1623 (20.9%)	1673 (21.6%)	
**Any Mastectomy**			0.064
No Mastectomy	2575 (33.2%)	2684 (34.6%)	
Mastectomy	5178 (66.8%)	5069 (65.4%)	
**Any Radiation**			0.062
No	4597 (59.3%)	4711 (60.8%)	
Yes	3156 (40.7%)	3042 (39.2%)	
**Any Chemotherapy**			0.495

	White (N = 7753)	Black (N = 7753)	p-value
No	5758 (74.3%)	5795 (74.7%)	
Yes	1995 (25.7%)	1958 (25.3%)	
**Any Doxorubicin**			0.383
No	6883 (88.8%)	6917 (89.2%)	
Yes	870 (11.2%)	836 (10.8%)	
**Any Taxane**			0.387
No	7259 (93.6%)	7285 (94.0%)	
Yes	494 (6.4%)	468 (6.0%)	
**Doxorubicin and BCT**			0.613
No	7587 (97.9%)	7596 (98.0%)	
Yes	166 (2.1%)	157 (2.0%)	
**Doxorubicin and Mastectomy**			0.168
No	7125 (91.9%)	7171 (92.5%)	
Yes	628 (8.1%)	582 (7.5%)	
**Doxorubicin and Radiation**			0.457
No	7276 (93.8%)	7298 (94.1%)	
Yes	477 (6.2%)	455 (5.9%)	
**Doxorubicin and Taxane**			0.398
No	7421 (95.7%)	7442 (96.0%)	
Yes	332 (4.3%)	311 (4.0%)	
**Taxane and BCT**			0.491
No	7671 (98.9%)	7662 (98.8%)	
Yes	82 (1.1%)	91 (1.2%)	
**Taxane and Mastectomy**			0.057
No	7426 (95.8%)	7472 (96.4%)	
Yes	327 (4.2%)	281 (3.6%)	
**Taxane and Radiation**			0.210
No	7461 (96.2%)	7490 (96.6%)	
Yes	292 (3.8%)	263 (3.4%)	
**Radiation and BCT**			0.470
No	6702 (86.4%)	6671 (86.0%)	
Yes	1051 (13.6%)	1082 (14.0%)	
**Radiation and Mastectomy**			0.002
No	5863 (75.6%)	6024 (77.7%)	
Yes	1890 (24.4%)	1729 (22.3%)	
**Radiation, Doxorubicin, Taxane**			0.239
No	7546 (97.3%)	7569 (97.6%)	
Yes	207 (2.7%)	184 (2.4%)	
**BCT, Doxorubicin, Taxane**			0.065
No	7709 (99.4%)	7690 (99.2%)	
Yes	44 (0.6%)	63 (0.8%)	
**BCT, Doxorubicin, Radiation**			0.611
No	7623 (98.3%)	7631 (98.4%)	
Yes	130 (1.7%)	122 (1.6%)	
**BCT, Taxane, Radiation**			0.315

	White (N = 7753)	Black (N = 7753)	p-value
No	7698 (99.3%)	7687 (99.1%)	
Yes	55 (0.7%)	66 (0.9%)	
**Mastectomy, Doxorubicin, Taxane**			0.027
No	7508 (96.8%)	7554 (97.4%)	
Yes	245 (3.2%)	199 (2.6%)	
**Mastectomy, Doxorubicin, Radiation**			0.212
No	7437 (95.9%)	7467 (96.3%)	
Yes	316 (4.1%)	286 (3.7%)	
**Mastectomy, Taxane, Radiation**			0.032
No	7554 (97.4%)	7594 (97.9%)	
Yes	199 (2.6%)	159 (2.1%)	
**Mastectomy, Doxorubicin, Taxane, Radiation**			0.007
No	7594 (97.9%)	7638 (98.5%)	
Yes	159 (2.1%)	115 (1.5%)	
**BCT, Doxorubicin, Taxane, Radiation**			0.058
No	7721 (99.6%)	7704 (99.4%)	
Yes	32 (0.4%)	49 (0.6%)	
**Any Breast Conserving Therapy (a)**			0.905
No BCT	7149 (92.2%)	7145 (92.2%)	
BCT	604 (7.8%)	608 (7.8%)	
**Any Mastectomy (a)**			0.743
No Mastectomy	1461 (18.8%)	1477 (19.1%)	
Mastectomy	6292 (81.2%)	6276 (80.9%)	
**Any Radiation (a)**			0.138
No	4513 (58.2%)	4604 (59.4%)	
Yes	3240 (41.8%)	3149 (40.6%)	
**Any Chemotherapy (a)**			0.123
No	5632 (72.6%)	5717 (73.7%)	
Yes	2121 (27.4%)	2036 (26.3%)	
**Any Doxorubicin**			0.383
No	6883 (88.8%)	6917 (89.2%)	
Yes	870 (11.2%)	836 (10.8%)	
**Any Taxane**			0.387
No	7259 (93.6%)	7285 (94.0%)	
Yes	494 (6.4%)	468 (6.0%)	
**Doxorubicin and BCT (a)**			0.308
No	7677 (99.0%)	7689 (99.2%)	
Yes	76 (1.0%)	64 (0.8%)	
**Doxorubicin and Mastectomy (a)**			0.453
No	7026 (90.6%)	7053 (91.0%)	
Yes	727 (9.4%)	700 (9.0%)	
**Doxorubicin and Radiation (a)**			0.404
No	7262 (93.7%)	7287 (94.0%)	
Yes	491 (6.3%)	466 (6.0%)	

	White (N = 7753)	Black (N = 7753)	p-value
**Doxorubicin and Taxane**			0.398
No	7421 (95.7%)	7442 (96.0%)	
Yes	332 (4.3%)	311 (4.0%)	
**Taxane and BCT (a)**			0.911
No	7713 (99.5%)	7712 (99.5%)	
Yes	40 (0.5%)	41 (0.5%)	
**Taxane and Mastectomy (a)**			0.211
No	7371 (95.1%)	7404 (95.5%)	
Yes	382 (4.9%)	349 (4.5%)	
**Taxane and Radiation (a)**			0.215
No	7455 (96.2%)	7484 (96.5%)	
Yes	298 (3.8%)	269 (3.5%)	
**Radiation and BCT (a)**			0.655
No	7432 (95.9%)	7443 (96.0%)	
Yes	321 (4.1%)	310 (4.0%)	
**Radiation and Mastectomy (a)**			0.238
No	5025 (64.8%)	5095 (65.7%)	
Yes	2728 (35.2%)	2658 (34.3%)	
**Radiation, Doxorubicin, and Taxane (a)**			0.243
No	7542 (97.3%)	7565 (97.6%)	
Yes	211 (2.7%)	188 (2.4%)	
**BCT, Doxorubicin, Taxane (a)**			0.752
No	7734 (99.8%)	7732 (99.7%)	
Yes	19 (0.2%)	21 (0.3%)	
**BCT, Doxorubicin, Radiation (a)**			0.263
No	7699 (99.3%)	7710 (99.4%)	
Yes	54 (0.7%)	43 (0.6%)	
**BCT, Taxane, Radiation (a)**			1.000
No	7731 (99.7%)	7731 (99.7%)	
Yes	22 (0.3%)	22 (0.3%)	
**Mastectomy, Doxorubicin, Taxane (a)**			0.286
No	7479 (96.5%)	7503 (96.8%)	
Yes	274 (3.5%)	250 (3.2%)	
**Mastectomy, Doxorubicin, Radiation (a)**			0.537
No	7345 (94.7%)	7362 (95.0%)	
Yes	408 (5.3%)	391 (5.0%)	
**Mastectomy, Taxane, Radiation (a)**			0.185
No	7509 (96.9%)	7537 (97.2%)	
Yes	244 (3.1%)	216 (2.8%)	
**Mastectomy, Doxorubicin, Taxane, Radiation (a)**			0.172
No	7569 (97.6%)	7594 (97.9%)	
Yes	184 (2.4%)	159 (2.1%)	
**BCT, Doxorubicin, Taxane, Radiation (a)**			0.841

	White (N = 7753)	Black (N = 7753)	p-value
No	7741 (99.8%)	7740 (99.8%)	
Yes	12 (0.2%)	13 (0.2%)	

Supplemental Table 2. Demographic, presentation, and treatment characteristics in the 2006-2013 sample after adjusting for the various sets of confounders. *Small cell values are obscured for privacy purposes.

Supplemental Table 2a. Controlling for Demographic Variables, 2006-2013

	White (N = 6186)	Black (N = 6186)	p-value
**Age**			0.474
Mean (SD)	75.46 (7.08)	75.56 (7.18)	
Median (Q1, Q3)	74.0 (70.0, 80.0)	74.0 (70.0, 80.0)	
**Size (mm)**			<0.001
Mean (SD)	21.22 (24.22)	26.12 (27.63)	
Median (Q1, Q3)	15.0 (10.0, 25.0)	20.0 (11.0, 32.0)	
**Number of Positive Nodes**			<0.001
Mean (SD)	0.87 (2.81)	1.09 (3.08)	
Median (Q1, Q3)	0.0 (0.0, 0.0)	0.0 (0.0, 1.0)	
**Number of Nodes Examined**			<0.001
Mean (SD)	5.08 (6.25)	5.61 (6.77)	
Median (Q1, Q3)	3.0 (1.0, 7.0)	3.0 (1.0, 9.0)	
**Year of Diagnosis**			1.000
2006	738 (11.9%)	743 (12.0%)	
2007	787 (12.7%)	783 (12.7%)	
2008	797 (12.9%)	791 (12.8%)	
2009	781 (12.6%)	779 (12.6%)	
2010	735 (11.9%)	738 (11.9%)	
2011	768 (12.4%)	777 (12.6%)	
2012	771 (12.5%)	767 (12.4%)	
2013	809 (13.1%)	808 (13.1%)	
**Registry**			1.000
Connecticut	207 (3.3%)	206 (3.3%)	
Detroit	874 (14.1%)	863 (14.0%)	
Hawaii	* (*)	* (*)	
Iowa	33 (0.5%)	34 (0.5%)	
New Mexico	* (*)	* (*)	
Seattle	92 (1.5%)	92 (1.5%)	
Utah	* (*)	* (*)	
Kentucky	260 (4.2%)	263 (4.3%)	
Louisiana	1039 (16.8%)	1045 (16.9%)	
New Jersey	1056 (17.1%)	1056 (17.1%)	
Georgia	1530 (24.7%)	1535 (24.8%)	
California	1068 (17.3%)	1066 (17.2%)	
**AJCC Stage**			<0.001
Stage I	3286 (53.1%)	2608 (42.2%)	
Stage II	1922 (31.1%)	2191 (35.4%)	
Stage III	595 (9.6%)	783 (12.7%)	
Stage IV	383 (6.2%)	604 (9.8%)	
**Tumor Grade**			<0.001
Grade I	1497 (24.2%)	1017 (16.4%)	

	White (N = 6186)	Black (N = 6186)	p-value
Grade II	2660 (43.0%)	2417 (39.1%)	
Grade III	1523 (24.6%)	2110 (34.1%)	
Grade IV	37 (0.6%)	48 (0.8%)	
Missing	469 (7.6%)	594 (9.6%)	
**ER Status**			<0.001
Negative	829 (13.4%)	1377 (22.3%)	
Positive	5019 (81.1%)	4413 (71.3%)	
Missing	338 (5.5%)	396 (6.4%)	
**PR Status**			<0.001
Negative	1563 (25.3%)	2146 (34.7%)	
Positive	4275 (69.1%)	3637 (58.8%)	
Missing	348 (5.6%)	403 (6.5%)	
**Tumor Size**			<0.001
0-.9cm	1397 (22.6%)	1096 (17.7%)	
1-1.9	2236 (36.1%)	1856 (30.0%)	
2-2.9	1139 (18.4%)	1226 (19.8%)	
3-3.9	541 (8.7%)	663 (10.7%)	
4+cm	729 (11.8%)	1111 (18.0%)	
Missing	144 (2.3%)	234 (3.8%)	
**Congestive Heart Failure**			<0.001
No	5425 (87.7%)	4936 (79.8%)	
Yes	761 (12.3%)	1250 (20.2%)	
**Past Arrhythmia**			<0.001
No	5538 (89.5%)	5398 (87.3%)	
Yes	648 (10.5%)	788 (12.7%)	
**Past Myocardial Infarction**			<0.001
No	5951 (96.2%)	5862 (94.8%)	
Yes	235 (3.8%)	324 (5.2%)	
**Angina**			0.055
No	6033 (97.5%)	5998 (97.0%)	
Yes	153 (2.5%)	188 (3.0%)	
**Valvular Heart Disease**			0.002
No	5278 (85.3%)	5394 (87.2%)	
Yes	908 (14.7%)	792 (12.8%)	
**Hypertension (uncomplicated)**			0.161
No	2221 (35.9%)	2296 (37.1%)	
Yes	3965 (64.1%)	3890 (62.9%)	
**Hypertension (complications)**			<0.001
No	5292 (85.5%)	4409 (71.3%)	
Yes	894 (14.5%)	1777 (28.7%)	
**Diabetes (uncomplicated)**			<0.001
No	4939 (79.8%)	4400 (71.1%)	
Yes	1247 (20.2%)	1786 (28.9%)	
**Diabetes (complications)**			<0.001
No	5722 (92.5%)	5043 (81.5%)	

	White (N = 6186)	Black (N = 6186)	p-value
Yes	464 (7.5%)	1143 (18.5%)	
**Kidney Disease**			<0.001
None	5650 (91.3%)	5027 (81.3%)	
Renal Dysfunction	48 (0.8%)	67 (1.1%)	
Renal Failure	488 (7.9%)	1092 (17.7%)	
**Liver disease**			0.770
No	6089 (98.4%)	6093 (98.5%)	
Yes	97 (1.6%)	93 (1.5%)	
**Chronic Lung Disease**			0.047
No	4929 (79.7%)	4839 (78.2%)	
Yes	1257 (20.3%)	1347 (21.8%)	
**Dementia**			<0.001
No	5852 (94.6%)	5653 (91.4%)	
Yes	334 (5.4%)	533 (8.6%)	
**Ischemic event**			0.026
No	6055 (97.9%)	6017 (97.3%)	
Yes	131 (2.1%)	169 (2.7%)	
**Stroke**			<0.001
No	6012 (97.2%)	5921 (95.7%)	
Yes	174 (2.8%)	265 (4.3%)	
**Hypothyroidism**			<0.001
No	4373 (70.7%)	5143 (83.1%)	
Yes	1813 (29.3%)	1043 (16.9%)	
**Paralysis**			<0.001
No	6055 (97.9%)	5912 (95.6%)	
Yes	131 (2.1%)	274 (4.4%)	
**Collagen Vascular Disease**			0.421
No	5813 (94.0%)	5834 (94.3%)	
Yes	373 (6.0%)	352 (5.7%)	
**Coagulopathy**			0.249
No	5866 (94.8%)	5837 (94.4%)	
Yes	320 (5.2%)	349 (5.6%)	
**Chronic Obstructive Pulmonary Disease**			0.018
No	4854 (78.5%)	4744 (76.7%)	
Yes	1332 (21.5%)	1442 (23.3%)	
**Peptic Ulcer Disease**			0.251
No	* (100%)	* (100%)	
Yes	* (0%)	* (0%)	
**Depression**			<0.001
No	5407 (87.4%)	5624 (90.9%)	
Yes	779 (12.6%)	562 (9.1%)	
**Cushing's Disease**			1.000
No	* (100%)	* (100%)	
Yes	* (0%)	* (0%)	
**Graves Disease**			.

	White (N = 6186)	Black (N = 6186)	p-value
No	6186 (100.0%)	6186 (100.0%)	
**Post-Inflammatory Pulmonary Fibrosis**			0.556
No	6111 (98.8%)	6118 (98.9%)	
Yes	75 (1.2%)	68 (1.1%)	
**Any Breast Conserving Therapy**			0.592
No BCT	5869 (94.9%)	5882 (95.1%)	
BCT	317 (5.1%)	304 (4.9%)	
**Any Mastectomy**			<0.001
No Mastectomy	929 (15.0%)	1387 (22.4%)	
Mastectomy	5257 (85.0%)	4799 (77.6%)	
**Any Radiation**			<0.001
No	2850 (46.1%)	3351 (54.2%)	
Yes	3336 (53.9%)	2835 (45.8%)	
**Any Chemotherapy**			<0.001
No	4792 (77.5%)	4586 (74.1%)	
Yes	1394 (22.5%)	1600 (25.9%)	
**Any Doxorubicin**			0.028
No	5726 (92.6%)	5660 (91.5%)	
Yes	460 (7.4%)	526 (8.5%)	
**Any Taxane**			0.005
No	5534 (89.5%)	5435 (87.9%)	
Yes	652 (10.5%)	751 (12.1%)	
**Doxorubicin and BCT**			0.277
No	6156 (99.5%)	6147 (99.4%)	
Yes	30 (0.5%)	39 (0.6%)	
**Doxorubicin and Mastectomy**			0.253
No	5808 (93.9%)	5777 (93.4%)	
Yes	378 (6.1%)	409 (6.6%)	
**Doxorubicin and Radiation**			0.670
No	5903 (95.4%)	5893 (95.3%)	
Yes	283 (4.6%)	293 (4.7%)	
**Doxorubicin and Taxane**			0.148
No	5896 (95.3%)	5861 (94.7%)	
Yes	290 (4.7%)	325 (5.3%)	
**Taxane and BCT**			0.627
No	6150 (99.4%)	6154 (99.5%)	
Yes	36 (0.6%)	32 (0.5%)	
**Taxane and Mastectomy**			0.271
No	5647 (91.3%)	5612 (90.7%)	
Yes	539 (8.7%)	574 (9.3%)	
**Taxane and Radiation**			0.971
No	5767 (93.2%)	5766 (93.2%)	
Yes	419 (6.8%)	420 (6.8%)	
**Radiation and BCT**			0.078
No	5964 (96.4%)	5999 (97.0%)	

	White (N = 6186)	Black (N = 6186)	p-value
Yes	222 (3.6%)	187 (3.0%)	
**Radiation and Mastectomy**			<0.001
No	3225 (52.1%)	3752 (60.7%)	
Yes	2961 (47.9%)	2434 (39.3%)	
**Radiation, Doxorubicin, Taxane**			0.723
No	5981 (96.7%)	5988 (96.8%)	
Yes	205 (3.3%)	198 (3.2%)	
**BCT, Doxorubicin, Taxane**			0.731
No	6168 (99.7%)	6170 (99.7%)	
Yes	18 (0.3%)	16 (0.3%)	
**BCT, Doxorubicin, Radiation**			0.207
No	6165 (99.7%)	6156 (99.5%)	
Yes	21 (0.3%)	30 (0.5%)	
**BCT, Taxane, Radiation**			0.668
No	6160 (99.6%)	6163 (99.6%)	
Yes	26 (0.4%)	23 (0.4%)	
**Mastectomy, Doxorubicin, Taxane**			0.519
No	5948 (96.2%)	5934 (95.9%)	
Yes	238 (3.8%)	252 (4.1%)	
**Mastectomy, Doxorubicin, Radiation**			0.741
No	5950 (96.2%)	5957 (96.3%)	
Yes	236 (3.8%)	229 (3.7%)	
**Mastectomy, Taxane, Radiation**			0.641
No	5828 (94.2%)	5840 (94.4%)	
Yes	358 (5.8%)	346 (5.6%)	
**Mastectomy, Doxorubicin, Taxane, Radiation**			0.372
No	6013 (97.2%)	6029 (97.5%)	
Yes	173 (2.8%)	157 (2.5%)	
**BCT, Doxorubicin, Taxane, Radiation**			1.000
No	6172 (99.8%)	6172 (99.8%)	
Yes	14 (0.2%)	14 (0.2%)	
**Any Breast Conserving Therapy (a)**			<0.001
No BCT	6062 (98.0%)	5990 (96.8%)	
BCT	124 (2.0%)	196 (3.2%)	
**Any Mastectomy (a)**			<0.001
No Mastectomy	689 (11.1%)	1182 (19.1%)	
Mastectomy	5497 (88.9%)	5004 (80.9%)	
**Any Radiation (a)**			<0.001
No	2804 (45.3%)	3287 (53.1%)	
Yes	3382 (54.7%)	2899 (46.9%)	
**Any Chemotherapy (a)**			0.045
No	4444 (71.8%)	4343 (70.2%)	
Yes	1742 (28.2%)	1843 (29.8%)	
**Any Doxorubicin**			0.028

	White (N = 6186)	Black (N = 6186)	p-value
No	5726 (92.6%)	5660 (91.5%)	
Yes	460 (7.4%)	526 (8.5%)	
**Any Taxane**			0.005
No	5534 (89.5%)	5435 (87.9%)	
Yes	652 (10.5%)	751 (12.1%)	
**Doxorubicin and BCT (a)**			0.161
No	* (100%)	* (100%)	
Yes	* (0%)	* (0%)	
**Doxorubicin and Mastectomy (a)**			0.164
No	5785 (93.5%)	5746 (92.9%)	
Yes	401 (6.5%)	440 (7.1%)	
**Doxorubicin and Radiation (a)**			0.644
No	5894 (95.3%)	5883 (95.1%)	
Yes	292 (4.7%)	303 (4.9%)	
**Doxorubicin and Taxane**			0.148
No	5896 (95.3%)	5861 (94.7%)	
Yes	290 (4.7%)	325 (5.3%)	
**Taxane and BCT (a)**			0.536
No	6167 (99.7%)	6163 (99.6%)	
Yes	19 (0.3%)	23 (0.4%)	
**Taxane and Mastectomy (a)**			0.167
No	5623 (90.9%)	5578 (90.2%)	
Yes	563 (9.1%)	608 (9.8%)	
**Taxane and Radiation (a)**			0.972
No	5758 (93.1%)	5757 (93.1%)	
Yes	428 (6.9%)	429 (6.9%)	
**Radiation and BCT (a)**			0.037
No	6131 (99.1%)	6107 (98.7%)	
Yes	55 (0.9%)	79 (1.3%)	
**Radiation and Mastectomy (a)**			<0.001
No	3007 (48.6%)	3567 (57.7%)	
Yes	3179 (51.4%)	2619 (42.3%)	
**Radiation, Doxorubicin, and Taxane (a)**			0.801
No	5979 (96.7%)	5984 (96.7%)	
Yes	207 (3.3%)	202 (3.3%)	
**BCT, Doxorubicin, Taxane (a)**			1.000
No	* (100%)	* (100%)	
Yes	* (0%)	* (0%)	
**BCT, Doxorubicin, Radiation (a)**			0.438
No	* (100%)	* (100%)	
Yes	* (0%)	* (0%)	
**BCT, Taxane, Radiation (a)**			0.371
No	* (100%)	* (100%)	
Yes	* (0%)	* (0%)	
**Mastectomy, Doxorubicin, Taxane (a)**			0.419

	White (N = 6186)	Black (N = 6186)	p-value
No	5936 (96.0%)	5918 (95.7%)	
Yes	250 (4.0%)	268 (4.3%)	
**Mastectomy, Doxorubicin, Radiation (a)**			0.929
No	5927 (95.8%)	5925 (95.8%)	
Yes	259 (4.2%)	261 (4.2%)	
**Mastectomy, Taxane, Radiation (a)**			0.881
No	5804 (93.8%)	5808 (93.9%)	
Yes	382 (6.2%)	378 (6.1%)	
**Mastectomy, Doxorubicin, Taxane, Radiation (a)**			0.591
No	6003 (97.0%)	6013 (97.2%)	
Yes	183 (3.0%)	173 (2.8%)	
**BCT, Doxorubicin, Taxane, Radiation (a)**			0.527
No	* (100%)	* (100%)	
Yes	* (0%)	* (0%)	

Supplemental Table 2b. Controlling for Demographic and Presentation Variables, 2006-2013

	White (N = 6186)	Black (N = 6186)	p-value
**Age**			0.253
Mean (SD)	75.70 (7.15)	75.56 (7.18)	
Median (Q1, Q3)	74.0 (70.0, 80.0)	74.0 (70.0, 80.0)	
**Size (mm)**			0.039
Mean (SD)	25.11 (25.51)	26.12 (27.63)	
Median (Q1, Q3)	19.0 (11.0, 30.0)	20.0 (11.0, 32.0)	
**Number of Positive Nodes**			0.474
Mean (SD)	1.13 (3.40)	1.09 (3.08)	
Median (Q1, Q3)	0.0 (0.0, 1.0)	0.0 (0.0, 1.0)	
**Number of Nodes Examined**			0.166
Mean (SD)	5.44 (6.79)	5.61 (6.77)	
Median (Q1, Q3)	2.0 (1.0, 8.0)	3.0 (1.0, 9.0)	
**Year of Diagnosis**			0.984
2006	754 (12.2%)	743 (12.0%)	
2007	773 (12.5%)	783 (12.7%)	
2008	811 (13.1%)	791 (12.8%)	
2009	793 (12.8%)	779 (12.6%)	
2010	735 (11.9%)	738 (11.9%)	
2011	788 (12.7%)	777 (12.6%)	
2012	760 (12.3%)	767 (12.4%)	
2013	772 (12.5%)	808 (13.1%)	
**Registry**			0.994
Connecticut	197 (3.2%)	206 (3.3%)	
Detroit	882 (14.3%)	863 (14.0%)	
Hawaii	* (*)	* (*)	

	White (N = 6186)	Black (N = 6186)	p-value
Iowa	32 (0.5%)	34 (0.5%)	
New Mexico	* (*)	* (*)	
Seattle	82 (1.3%)	92 (1.5%)	
Utah	* (*)	* (*)	
Kentucky	276 (4.5%)	263 (4.3%)	
Louisiana	1018 (16.5%)	1045 (16.9%)	
New Jersey	1073 (17.3%)	1056 (17.1%)	
Georgia	1530 (24.7%)	1535 (24.8%)	
California	1069 (17.3%)	1066 (17.2%)	
**AJCC Stage**			0.431
Stage I	2673 (43.2%)	2608 (42.2%)	
Stage II	2104 (34.0%)	2191 (35.4%)	
Stage III	799 (12.9%)	783 (12.7%)	
Stage IV	610 (9.9%)	604 (9.8%)	
**Tumor Grade**			0.812
Grade I	1044 (16.9%)	1017 (16.4%)	
Grade II	2408 (38.9%)	2417 (39.1%)	
Grade III	2077 (33.6%)	2110 (34.1%)	
Grade IV	41 (0.7%)	48 (0.8%)	
Missing	616 (10.0%)	594 (9.6%)	
**ER Status**			0.819
Negative	1367 (22.1%)	1377 (22.3%)	
Positive	4406 (71.2%)	4413 (71.3%)	
Missing	413 (6.7%)	396 (6.4%)	
**PR Status**			0.777
Negative	2129 (34.4%)	2146 (34.7%)	
Positive	3635 (58.8%)	3637 (58.8%)	
Missing	422 (6.8%)	403 (6.5%)	
**Tumor Size**			0.660
0-.9cm	1117 (18.1%)	1096 (17.7%)	
1-1.9	1921 (31.1%)	1856 (30.0%)	
2-2.9	1183 (19.1%)	1226 (19.8%)	
3-3.9	651 (10.5%)	663 (10.7%)	
4+cm	1069 (17.3%)	1111 (18.0%)	
Missing	245 (4.0%)	234 (3.8%)	
**Congestive Heart Failure**			0.544
No	4963 (80.2%)	4936 (79.8%)	
Yes	1223 (19.8%)	1250 (20.2%)	
**Past Arrhythmia**			0.533
No	5421 (87.6%)	5398 (87.3%)	
Yes	765 (12.4%)	788 (12.7%)	
**Past Myocardial Infarction**			0.404
No	5841 (94.4%)	5862 (94.8%)	
Yes	345 (5.6%)	324 (5.2%)	
**Angina**			0.875

	White (N = 6186)	Black (N = 6186)	p-value
No	6001 (97.0%)	5998 (97.0%)	
Yes	185 (3.0%)	188 (3.0%)	
**Valvular Heart Disease**			0.685
No	5409 (87.4%)	5394 (87.2%)	
Yes	777 (12.6%)	792 (12.8%)	
**Hypertension (uncomplicated)**			0.502
No	2260 (36.5%)	2296 (37.1%)	
Yes	3926 (63.5%)	3890 (62.9%)	
**Hypertension (complications)**			0.619
No	4434 (71.7%)	4409 (71.3%)	
Yes	1752 (28.3%)	1777 (28.7%)	
**Diabetes (uncomplicated)**			0.621
No	4375 (70.7%)	4400 (71.1%)	
Yes	1811 (29.3%)	1786 (28.9%)	
**Diabetes (complications)**			0.530
No	5070 (82.0%)	5043 (81.5%)	
Yes	1116 (18.0%)	1143 (18.5%)	
**Kidney Disease**			0.955
None	5023 (81.2%)	5027 (81.3%)	
Renal Dysfunction	64 (1.0%)	67 (1.1%)	
Renal Failure	1099 (17.8%)	1092 (17.7%)	
**Liver disease**			0.598
No	6100 (98.6%)	6093 (98.5%)	
Yes	86 (1.4%)	93 (1.5%)	
**Chronic Lung Disease**			0.587
No	4814 (77.8%)	4839 (78.2%)	
Yes	1372 (22.2%)	1347 (21.8%)	
**Dementia**			0.872
No	5658 (91.5%)	5653 (91.4%)	
Yes	528 (8.5%)	533 (8.6%)	
**Ischemic event**			0.781
No	6022 (97.3%)	6017 (97.3%)	
Yes	164 (2.7%)	169 (2.7%)	
**Stroke**			0.859
No	5925 (95.8%)	5921 (95.7%)	
Yes	261 (4.2%)	265 (4.3%)	
**Hypothyroidism**			0.867
No	5136 (83.0%)	5143 (83.1%)	
Yes	1050 (17.0%)	1043 (16.9%)	
**Paralysis**			0.507
No	5927 (95.8%)	5912 (95.6%)	
Yes	259 (4.2%)	274 (4.4%)	
**Collagen Vascular Disease**			0.907
No	5831 (94.3%)	5834 (94.3%)	
Yes	355 (5.7%)	352 (5.7%)	

	White (N = 6186)	Black (N = 6186)	p-value
**Coagulopathy**			0.503
No	5854 (94.6%)	5837 (94.4%)	
Yes	332 (5.4%)	349 (5.6%)	
**Chronic Obstructive Pulmonary Disease**			0.641
No	4722 (76.3%)	4744 (76.7%)	
Yes	1464 (23.7%)	1442 (23.3%)	
**Peptic Ulcer Disease**			0.841
No	6173 (99.8%)	6174 (99.8%)	
Yes	13 (0.2%)	12 (0.2%)	
**Depression**			0.706
No	5636 (91.1%)	5624 (90.9%)	
Yes	550 (8.9%)	562 (9.1%)	
**Cushing's Disease**			0.655
No	* (100%)	* (100%)	
Yes	* (0%)	* (0%)	
**Graves Disease**			.
No	6186 (100.0%)	6186 (100.0%)	
**Post-Inflammatory Pulmonary Fibrosis**			0.864
No	6116 (98.9%)	6118 (98.9%)	
Yes	70 (1.1%)	68 (1.1%)	
**Any Breast Conserving Therapy**			0.436
No BCT	5863 (94.8%)	5882 (95.1%)	
BCT	323 (5.2%)	304 (4.9%)	
**Any Mastectomy**			<0.001
No Mastectomy	1189 (19.2%)	1387 (22.4%)	
Mastectomy	4997 (80.8%)	4799 (77.6%)	
**Any Radiation**			<0.001
No	3100 (50.1%)	3351 (54.2%)	
Yes	3086 (49.9%)	2835 (45.8%)	
**Any Chemotherapy**			0.117
No	4509 (72.9%)	4586 (74.1%)	
Yes	1677 (27.1%)	1600 (25.9%)	
**Any Doxorubicin**			0.146
No	5614 (90.8%)	5660 (91.5%)	
Yes	572 (9.2%)	526 (8.5%)	
**Any Taxane**			0.066
No	5367 (86.8%)	5435 (87.9%)	
Yes	819 (13.2%)	751 (12.1%)	
**Doxorubicin and BCT**			0.285
No	6137 (99.2%)	6147 (99.4%)	
Yes	49 (0.8%)	39 (0.6%)	
**Doxorubicin and Mastectomy**			0.255
No	5745 (92.9%)	5777 (93.4%)	
Yes	441 (7.1%)	409 (6.6%)	
**Doxorubicin and Radiation**			0.006

	White (N = 6186)	Black (N = 6186)	p-value
No	5824 (94.1%)	5893 (95.3%)	
Yes	362 (5.9%)	293 (4.7%)	
**Doxorubicin and Taxane**			0.100
No	5819 (94.1%)	5861 (94.7%)	
Yes	367 (5.9%)	325 (5.3%)	
**Taxane and BCT**			0.058
No	6137 (99.2%)	6154 (99.5%)	
Yes	49 (0.8%)	32 (0.5%)	
**Taxane and Mastectomy**			0.079
No	5554 (89.8%)	5612 (90.7%)	
Yes	632 (10.2%)	574 (9.3%)	
**Taxane and Radiation**			0.007
No	5687 (91.9%)	5766 (93.2%)	
Yes	499 (8.1%)	420 (6.8%)	
**Radiation and BCT**			0.356
No	5981 (96.7%)	5999 (97.0%)	
Yes	205 (3.3%)	187 (3.0%)	
**Radiation and Mastectomy**			<0.001
No	3545 (57.3%)	3752 (60.7%)	
Yes	2641 (42.7%)	2434 (39.3%)	
**Radiation, Doxorubicin, Taxane**			0.008
No	5933 (95.9%)	5988 (96.8%)	
Yes	253 (4.1%)	198 (3.2%)	
**BCT, Doxorubicin, Taxane**			0.159
No	6161 (99.6%)	6170 (99.7%)	
Yes	25 (0.4%)	16 (0.3%)	
**BCT, Doxorubicin, Radiation**			0.391
No	6149 (99.4%)	6156 (99.5%)	
Yes	37 (0.6%)	30 (0.5%)	
**BCT, Taxane, Radiation**			0.335
No	6156 (99.5%)	6163 (99.6%)	
Yes	30 (0.5%)	23 (0.4%)	
**Mastectomy, Doxorubicin, Taxane**			0.171
No	5903 (95.4%)	5934 (95.9%)	
Yes	283 (4.6%)	252 (4.1%)	
**Mastectomy, Doxorubicin, Radiation**			0.015
No	5903 (95.4%)	5957 (96.3%)	
Yes	283 (4.6%)	229 (3.7%)	
**Mastectomy, Taxane, Radiation**			0.013
No	5774 (93.3%)	5840 (94.4%)	
Yes	412 (6.7%)	346 (5.6%)	
**Mastectomy, Doxorubicin, Taxane, Radiation**			0.016
No	5984 (96.7%)	6029 (97.5%)	
Yes	202 (3.3%)	157 (2.5%)	

	White (N = 6186)	Black (N = 6186)	p-value
**BCT, Doxorubicin, Taxane, Radiation**			0.479
No	6168 (99.7%)	6172 (99.8%)	
Yes	18 (0.3%)	14 (0.2%)	
**Any Breast Conserving Therapy (a)**			0.345
No BCT	6008 (97.1%)	5990 (96.8%)	
BCT	178 (2.9%)	196 (3.2%)	
**Any Mastectomy (a)**			<0.001
No Mastectomy	975 (15.8%)	1182 (19.1%)	
Mastectomy	5211 (84.2%)	5004 (80.9%)	
**Any Radiation (a)**			<0.001
No	3024 (48.9%)	3287 (53.1%)	
Yes	3162 (51.1%)	2899 (46.9%)	
**Any Chemotherapy (a)**			0.009
No	4208 (68.0%)	4343 (70.2%)	
Yes	1978 (32.0%)	1843 (29.8%)	
**Any Doxorubicin**			0.146
No	5614 (90.8%)	5660 (91.5%)	
Yes	572 (9.2%)	526 (8.5%)	
**Any Taxane**			0.066
No	5367 (86.8%)	5435 (87.9%)	
Yes	819 (13.2%)	751 (12.1%)	
**Doxorubicin and BCT (a)**			0.410
No	6165 (99.7%)	6170 (99.7%)	
Yes	21 (0.3%)	16 (0.3%)	
**Doxorubicin and Mastectomy (a)**			0.256
No	5713 (92.4%)	5746 (92.9%)	
Yes	473 (7.6%)	440 (7.1%)	
**Doxorubicin and Radiation (a)**			0.007
No	5815 (94.0%)	5883 (95.1%)	
Yes	371 (6.0%)	303 (4.9%)	
**Doxorubicin and Taxane**			0.100
No	5819 (94.1%)	5861 (94.7%)	
Yes	367 (5.9%)	325 (5.3%)	
**Taxane and BCT (a)**			0.404
No	6157 (99.5%)	6163 (99.6%)	
Yes	29 (0.5%)	23 (0.4%)	
**Taxane and Mastectomy (a)**			0.130
No	5527 (89.3%)	5578 (90.2%)	
Yes	659 (10.7%)	608 (9.8%)	
**Taxane and Radiation (a)**			0.006
No	5676 (91.8%)	5757 (93.1%)	
Yes	510 (8.2%)	429 (6.9%)	
**Radiation and BCT (a)**			0.808
No	6110 (98.8%)	6107 (98.7%)	
Yes	76 (1.2%)	79 (1.3%)	

	White (N = 6186)	Black (N = 6186)	p-value
**Radiation and Mastectomy (a)**			<0.001
No	3332 (53.9%)	3567 (57.7%)	
Yes	2854 (46.1%)	2619 (42.3%)	
**Radiation, Doxorubicin, and Taxane (a)**			0.010
No	5930 (95.9%)	5984 (96.7%)	
Yes	256 (4.1%)	202 (3.3%)	
**BCT, Doxorubicin, Taxane (a)**			0.126
No	* (100%)	* (100%)	
Yes	* (0%)	* (0%)	
**BCT, Doxorubicin, Radiation (a)**			0.393
No	* (100%)	* (100%)	
Yes	* (0%)	* (0%)	
**BCT, Taxane, Radiation (a)**			0.200
No	* (100%)	* (100%)	
Yes	* (0%)	* (0%)	
**Mastectomy, Doxorubicin, Taxane (a)**			0.227
No	5890 (95.2%)	5918 (95.7%)	
Yes	296 (4.8%)	268 (4.3%)	
**Mastectomy, Doxorubicin, Radiation (a)**			0.019
No	5870 (94.9%)	5925 (95.8%)	
Yes	316 (5.1%)	261 (4.2%)	
**Mastectomy, Taxane, Radiation (a)**			0.027
No	5747 (92.9%)	5808 (93.9%)	
Yes	439 (7.1%)	378 (6.1%)	
**Mastectomy, Doxorubicin, Taxane, Radiation (a)**			0.034
No	5972 (96.5%)	6013 (97.2%)	
Yes	214 (3.5%)	173 (2.8%)	
**BCT, Doxorubicin, Taxane, Radiation (a)**			0.109
No	* (100%)	* (100%)	
Yes	* (0%)	* (0%)	

Supplemental Table 2c. Controlling for Demographic, Presentation, and Treatment Variables, 2006-2013

	White (N = 6186)	Black (N = 6186)	p-value
**Age**			0.736
Mean (SD)	75.60 (7.16)	75.56 (7.18)	
Median (Q1, Q3)	74.0 (70.0, 81.0)	74.0 (70.0, 80.0)	
**Size (mm)**			0.380
Mean (SD)	25.65 (29.91)	26.12 (27.63)	
Median (Q1, Q3)	20.0 (11.0, 31.0)	20.0 (11.0, 32.0)	
**Number of Positive Nodes**			0.941
Mean (SD)	1.08 (3.02)	1.09 (3.08)	
Median (Q1, Q3)	0.0 (0.0, 1.0)	0.0 (0.0, 1.0)	

	White (N = 6186)	Black (N = 6186)	p-value
**Number of Nodes Examined**			0.830
Mean (SD)	5.64 (6.75)	5.61 (6.77)	
Median (Q1, Q3)	3.0 (1.0, 9.0)	3.0 (1.0, 9.0)	
**Year of Diagnosis**			0.925
2006	767 (12.4%)	743 (12.0%)	
2007	798 (12.9%)	783 (12.7%)	
2008	762 (12.3%)	791 (12.8%)	
2009	774 (12.5%)	779 (12.6%)	
2010	769 (12.4%)	738 (11.9%)	
2011	768 (12.4%)	777 (12.6%)	
2012	774 (12.5%)	767 (12.4%)	
2013	774 (12.5%)	808 (13.1%)	
**Registry**			0.999
Connecticut	210 (3.4%)	206 (3.3%)	
Detroit	871 (14.1%)	863 (14.0%)	
Hawaii	* (*)	* (*)	
Iowa	32 (0.5%)	34 (0.5%)	
New Mexico	* (*)	* (*)	
Seattle	90 (1.5%)	92 (1.5%)	
Utah	* (*)	* (*)	
Kentucky	273 (4.4%)	263 (4.3%)	
Louisiana	1035 (16.7%)	1045 (16.9%)	
New Jersey	1055 (17.1%)	1056 (17.1%)	
Georgia	1551 (25.1%)	1535 (24.8%)	
California	1042 (16.8%)	1066 (17.2%)	
**AJCC Stage**			0.993
Stage I	2619 (42.3%)	2608 (42.2%)	
Stage II	2191 (35.4%)	2191 (35.4%)	
Stage III	773 (12.5%)	783 (12.7%)	
Stage IV	603 (9.7%)	604 (9.8%)	
**Tumor Grade**			0.876
Grade I	993 (16.1%)	1017 (16.4%)	
Grade II	2450 (39.6%)	2417 (39.1%)	
Grade III	2087 (33.7%)	2110 (34.1%)	
Grade IV	43 (0.7%)	48 (0.8%)	
Missing	613 (9.9%)	594 (9.6%)	
**ER Status**			0.488
Negative	1326 (21.4%)	1377 (22.3%)	
Positive	4472 (72.3%)	4413 (71.3%)	
Missing	388 (6.3%)	396 (6.4%)	
**PR Status**			0.815
Negative	2117 (34.2%)	2146 (34.7%)	
Positive	3672 (59.4%)	3637 (58.8%)	
Missing	397 (6.4%)	403 (6.5%)	
**Tumor Size**			0.999

	White (N = 6186)	Black (N = 6186)	p-value
0-.9cm	1111 (18.0%)	1096 (17.7%)	
1-1.9	1836 (29.7%)	1856 (30.0%)	
2-2.9	1225 (19.8%)	1226 (19.8%)	
3-3.9	663 (10.7%)	663 (10.7%)	
4+cm	1113 (18.0%)	1111 (18.0%)	
Missing	238 (3.8%)	234 (3.8%)	
**Congestive Heart Failure**			0.214
No	4991 (80.7%)	4936 (79.8%)	
Yes	1195 (19.3%)	1250 (20.2%)	
**Past Arrhythmia**			0.914
No	5394 (87.2%)	5398 (87.3%)	
Yes	792 (12.8%)	788 (12.7%)	
**Past Myocardial Infarction**			0.389
No	5883 (95.1%)	5862 (94.8%)	
Yes	303 (4.9%)	324 (5.2%)	
**Angina**			0.672
No	6006 (97.1%)	5998 (97.0%)	
Yes	180 (2.9%)	188 (3.0%)	
**Valvular Heart Disease**			0.432
No	5423 (87.7%)	5394 (87.2%)	
Yes	763 (12.3%)	792 (12.8%)	
**Hypertension (uncomplicated)**			0.112
No	2211 (35.7%)	2296 (37.1%)	
Yes	3975 (64.3%)	3890 (62.9%)	
**Hypertension (complications)**			0.082
No	4496 (72.7%)	4409 (71.3%)	
Yes	1690 (27.3%)	1777 (28.7%)	
**Diabetes (uncomplicated)**			0.269
No	4344 (70.2%)	4400 (71.1%)	
Yes	1842 (29.8%)	1786 (28.9%)	
**Diabetes (complications)**			0.561
No	5068 (81.9%)	5043 (81.5%)	
Yes	1118 (18.1%)	1143 (18.5%)	
**Kidney Disease**			0.358
None	5085 (82.2%)	5027 (81.3%)	
Renal Dysfunction	69 (1.1%)	67 (1.1%)	
Renal Failure	1032 (16.7%)	1092 (17.7%)	
**Liver disease**			0.612
No	6086 (98.4%)	6093 (98.5%)	
Yes	100 (1.6%)	93 (1.5%)	
**Chronic Lung Disease**			0.498
No	4870 (78.7%)	4839 (78.2%)	
Yes	1316 (21.3%)	1347 (21.8%)	
**Dementia**			0.629
No	5668 (91.6%)	5653 (91.4%)	

	White (N = 6186)	Black (N = 6186)	p-value
Yes	518 (8.4%)	533 (8.6%)	
**Ischemic event**			0.189
No	6040 (97.6%)	6017 (97.3%)	
Yes	146 (2.4%)	169 (2.7%)	
**Stroke**			0.237
No	5947 (96.1%)	5921 (95.7%)	
Yes	239 (3.9%)	265 (4.3%)	
**Hypothyroidism**			0.848
No	5135 (83.0%)	5143 (83.1%)	
Yes	1051 (17.0%)	1043 (16.9%)	
**Paralysis**			0.137
No	5945 (96.1%)	5912 (95.6%)	
Yes	241 (3.9%)	274 (4.4%)	
**Collagen Vascular Disease**			0.617
No	5821 (94.1%)	5834 (94.3%)	
Yes	365 (5.9%)	352 (5.7%)	
**Coagulopathy**			0.938
No	5835 (94.3%)	5837 (94.4%)	
Yes	351 (5.7%)	349 (5.6%)	
**Chronic Obstructive Pulmonary Disease**			0.393
No	4784 (77.3%)	4744 (76.7%)	
Yes	1402 (22.7%)	1442 (23.3%)	
**Peptic Ulcer Disease**			0.449
No	* (100%)	* (100%)	
Yes	* (0%)	* (0%)	
**Depression**			0.950
No	5626 (90.9%)	5624 (90.9%)	
Yes	560 (9.1%)	562 (9.1%)	
**Cushing's Disease**			0.564
No	* (100%)	* (100%)	
Yes	* (0%)	* (0%)	
**Graves Disease**			.
No	6186 (100.0%)	6186 (100.0%)	
**Post-Inflammatory Pulmonary Fibrosis**			0.794
No	6121 (98.9%)	6118 (98.9%)	
Yes	65 (1.1%)	68 (1.1%)	
**Any Breast Conserving Therapy**			0.804
No BCT	5876 (95.0%)	5882 (95.1%)	
BCT	310 (5.0%)	304 (4.9%)	
**Any Mastectomy**			0.829
No Mastectomy	1377 (22.3%)	1387 (22.4%)	
Mastectomy	4809 (77.7%)	4799 (77.6%)	
**Any Radiation**			0.971
No	3349 (54.1%)	3351 (54.2%)	
Yes	2837 (45.9%)	2835 (45.8%)	

	White (N = 6186)	Black (N = 6186)	p-value
**Any Chemotherapy**			0.774
No	4572 (73.9%)	4586 (74.1%)	
Yes	1614 (26.1%)	1600 (25.9%)	
**Any Doxorubicin**			0.974
No	5659 (91.5%)	5660 (91.5%)	
Yes	527 (8.5%)	526 (8.5%)	
**Any Taxane**			0.471
No	5461 (88.3%)	5435 (87.9%)	
Yes	725 (11.7%)	751 (12.1%)	
**Doxorubicin and BCT**			0.818
No	6149 (99.4%)	6147 (99.4%)	
Yes	37 (0.6%)	39 (0.6%)	
**Doxorubicin and Mastectomy**			0.692
No	5766 (93.2%)	5777 (93.4%)	
Yes	420 (6.8%)	409 (6.6%)	
**Doxorubicin and Radiation**			0.530
No	5878 (95.0%)	5893 (95.3%)	
Yes	308 (5.0%)	293 (4.7%)	
**Doxorubicin and Taxane**			0.777
No	5868 (94.9%)	5861 (94.7%)	
Yes	318 (5.1%)	325 (5.3%)	
**Taxane and BCT**			0.546
No	6149 (99.4%)	6154 (99.5%)	
Yes	37 (0.6%)	32 (0.5%)	
**Taxane and Mastectomy**			0.709
No	5624 (90.9%)	5612 (90.7%)	
Yes	562 (9.1%)	574 (9.3%)	
**Taxane and Radiation**			0.458
No	5745 (92.9%)	5766 (93.2%)	
Yes	441 (7.1%)	420 (6.8%)	
**Radiation and BCT**			0.361
No	6016 (97.3%)	5999 (97.0%)	
Yes	170 (2.7%)	187 (3.0%)	
**Radiation and Mastectomy**			0.854
No	3742 (60.5%)	3752 (60.7%)	
Yes	2444 (39.5%)	2434 (39.3%)	
**Radiation, Doxorubicin, Taxane**			0.614
No	5978 (96.6%)	5988 (96.8%)	
Yes	208 (3.4%)	198 (3.2%)	
**BCT, Doxorubicin, Taxane**			1.000
No	6170 (99.7%)	6170 (99.7%)	
Yes	16 (0.3%)	16 (0.3%)	
**BCT, Doxorubicin, Radiation**			0.413
No	6162 (99.6%)	6156 (99.5%)	
Yes	24 (0.4%)	30 (0.5%)	

	White (N = 6186)	Black (N = 6186)	p-value
**BCT, Taxane, Radiation**			1.000
No	6163 (99.6%)	6163 (99.6%)	
Yes	23 (0.4%)	23 (0.4%)	
**Mastectomy, Doxorubicin, Taxane**			1.000
No	5934 (95.9%)	5934 (95.9%)	
Yes	252 (4.1%)	252 (4.1%)	
**Mastectomy, Doxorubicin, Radiation**			0.265
No	5933 (95.9%)	5957 (96.3%)	
Yes	253 (4.1%)	229 (3.7%)	
**Mastectomy, Taxane, Radiation**			0.463
No	5821 (94.1%)	5840 (94.4%)	
Yes	365 (5.9%)	346 (5.6%)	
**Mastectomy, Doxorubicin, Taxane, Radiation**			0.501
No	6017 (97.3%)	6029 (97.5%)	
Yes	169 (2.7%)	157 (2.5%)	
**BCT, Doxorubicin, Taxane, Radiation**			0.548
No	* (100%)	* (100%)	
Yes	* (0%)	* (0%)	
**Any Breast Conserving Therapy (a)**			0.077
No BCT	6023 (97.4%)	5990 (96.8%)	
BCT	163 (2.6%)	196 (3.2%)	
**Any Mastectomy (a)**			0.421
No Mastectomy	1147 (18.5%)	1182 (19.1%)	
Mastectomy	5039 (81.5%)	5004 (80.9%)	
**Any Radiation (a)**			0.705
No	3266 (52.8%)	3287 (53.1%)	
Yes	2920 (47.2%)	2899 (46.9%)	
**Any Chemotherapy (a)**			0.132
No	4266 (69.0%)	4343 (70.2%)	
Yes	1920 (31.0%)	1843 (29.8%)	
**Any Doxorubicin**			0.974
No	5659 (91.5%)	5660 (91.5%)	
Yes	527 (8.5%)	526 (8.5%)	
**Any Taxane**			0.471
No	5461 (88.3%)	5435 (87.9%)	
Yes	725 (11.7%)	751 (12.1%)	
**Doxorubicin and BCT (a)**			0.161
No	* (100%)	* (100%)	
Yes	* (0%)	* (0%)	
**Doxorubicin and Mastectomy (a)**			0.603
No	5731 (92.6%)	5746 (92.9%)	
Yes	455 (7.4%)	440 (7.1%)	
**Doxorubicin and Radiation (a)**			0.510
No	5867 (94.8%)	5883 (95.1%)	

	White (N = 6186)	Black (N = 6186)	p-value
Yes	319 (5.2%)	303 (4.9%)	
**Doxorubicin and Taxane**			0.777
No	5868 (94.9%)	5861 (94.7%)	
Yes	318 (5.1%)	325 (5.3%)	
**Taxane and BCT (a)**			0.262
No	6170 (99.7%)	6163 (99.6%)	
Yes	16 (0.3%)	23 (0.4%)	
**Taxane and Mastectomy (a)**			0.649
No	5593 (90.4%)	5578 (90.2%)	
Yes	593 (9.6%)	608 (9.8%)	
**Taxane and Radiation (a)**			0.402
No	5733 (92.7%)	5757 (93.1%)	
Yes	453 (7.3%)	429 (6.9%)	
**Radiation and BCT (a)**			0.010
No	6136 (99.2%)	6107 (98.7%)	
Yes	50 (0.8%)	79 (1.3%)	
**Radiation and Mastectomy (a)**			0.573
No	3536 (57.2%)	3567 (57.7%)	
Yes	2650 (42.8%)	2619 (42.3%)	
**Radiation, Doxorubicin, and Taxane (a)**			0.617
No	5974 (96.6%)	5984 (96.7%)	
Yes	212 (3.4%)	202 (3.3%)	
**BCT, Doxorubicin, Taxane (a)**			0.206
No	* (100%)	* (100%)	
Yes	* (0%)	* (0%)	
**BCT, Doxorubicin, Radiation (a)**			0.083
No	* (100%)	* (100%)	
Yes	* (0%)	* (0%)	
**BCT, Taxane, Radiation (a)**			0.405
No	* (100%)	* (100%)	
Yes	* (0%)	* (0%)	
**Mastectomy, Doxorubicin, Taxane (a)**			0.965
No	5917 (95.7%)	5918 (95.7%)	
Yes	269 (4.3%)	268 (4.3%)	
**Mastectomy, Doxorubicin, Radiation (a)**			0.314
No	5902 (95.4%)	5925 (95.8%)	
Yes	284 (4.6%)	261 (4.2%)	
**Mastectomy, Taxane, Radiation (a)**			0.481
No	5789 (93.6%)	5808 (93.9%)	
Yes	397 (6.4%)	378 (6.1%)	
**Mastectomy, Doxorubicin, Taxane, Radiation (a)**			0.591
No	6003 (97.0%)	6013 (97.2%)	
Yes	183 (3.0%)	173 (2.8%)	

	White (N = 6186)	Black (N = 6186)	p-value
**BCT, Doxorubicin, Taxane, Radiation (a)**			0.180
No	* (100%)	* (100%)	
Yes	* (0%)	* (0%)	

Supplemental Table 2d. Controlling for Demographic, Presentation, and Augmented Treatment Variables (a = Augmented Definitions), 2006-2013

	White (N = 6186)	Black (N = 6186)	p-value
**Age**			0.399
Mean (SD)	75.67 (7.17)	75.56 (7.18)	
Median (Q1, Q3)	74.0 (70.0, 81.0)	74.0 (70.0, 80.0)	
**Size (mm)**			0.208
Mean (SD)	25.48 (27.60)	26.12 (27.63)	
Median (Q1, Q3)	20.0 (11.0, 32.0)	20.0 (11.0, 32.0)	
**Number of Positive Nodes**			0.859
Mean (SD)	1.08 (3.09)	1.09 (3.08)	
Median (Q1, Q3)	0.0 (0.0, 1.0)	0.0 (0.0, 1.0)	
**Number of Nodes Examined**			0.182
Mean (SD)	5.45 (6.67)	5.61 (6.77)	
Median (Q1, Q3)	3.0 (1.0, 9.0)	3.0 (1.0, 9.0)	
**Year of Diagnosis**			0.532
2006	698 (11.3%)	743 (12.0%)	
2007	732 (11.8%)	783 (12.7%)	
2008	850 (13.7%)	791 (12.8%)	
2009	802 (13.0%)	779 (12.6%)	
2010	733 (11.8%)	738 (11.9%)	
2011	802 (13.0%)	777 (12.6%)	
2012	757 (12.2%)	767 (12.4%)	
2013	812 (13.1%)	808 (13.1%)	
**Registry**			0.911
Connecticut	215 (3.5%)	206 (3.3%)	
Detroit	855 (13.8%)	863 (14.0%)	
Hawaii	* (*)	* (*)	
Iowa	34 (0.5%)	34 (0.5%)	
New Mexico	* (*)	* (*)	
Seattle	78 (1.3%)	92 (1.5%)	
Utah	* (*)	* (*)	
Kentucky	281 (4.5%)	263 (4.3%)	
Louisiana	1005 (16.2%)	1045 (16.9%)	
New Jersey	1089 (17.6%)	1056 (17.1%)	
Georgia	1533 (24.8%)	1535 (24.8%)	
California	1064 (17.2%)	1066 (17.2%)	
**AJCC Stage**			0.975
Stage I	2632 (42.5%)	2608 (42.2%)	
Stage II	2180 (35.2%)	2191 (35.4%)	
Stage III	772 (12.5%)	783 (12.7%)	

	White (N = 6186)	Black (N = 6186)	p-value
Stage IV	602 (9.7%)	604 (9.8%)	
**Tumor Grade**			0.865
Grade I	1050 (17.0%)	1017 (16.4%)	
Grade II	2371 (38.3%)	2417 (39.1%)	
Grade III	2110 (34.1%)	2110 (34.1%)	
Grade IV	45 (0.7%)	48 (0.8%)	
Missing	610 (9.9%)	594 (9.6%)	
**ER Status**			0.933
Negative	1382 (22.3%)	1377 (22.3%)	
Positive	4418 (71.4%)	4413 (71.3%)	
Missing	386 (6.2%)	396 (6.4%)	
**PR Status**			0.967
Negative	2151 (34.8%)	2146 (34.7%)	
Positive	3639 (58.8%)	3637 (58.8%)	
Missing	396 (6.4%)	403 (6.5%)	
**Tumor Size**			0.865
0-.9cm	1069 (17.3%)	1096 (17.7%)	
1-1.9	1855 (30.0%)	1856 (30.0%)	
2-2.9	1231 (19.9%)	1226 (19.8%)	
3-3.9	699 (11.3%)	663 (10.7%)	
4+cm	1086 (17.6%)	1111 (18.0%)	
Missing	246 (4.0%)	234 (3.8%)	
**Congestive Heart Failure**			0.858
No	4944 (79.9%)	4936 (79.8%)	
Yes	1242 (20.1%)	1250 (20.2%)	
**Past Arrhythmia**			0.978
No	5397 (87.2%)	5398 (87.3%)	
Yes	789 (12.8%)	788 (12.7%)	
**Past Myocardial Infarction**			0.303
No	5836 (94.3%)	5862 (94.8%)	
Yes	350 (5.7%)	324 (5.2%)	
**Angina**			0.536
No	5986 (96.8%)	5998 (97.0%)	
Yes	200 (3.2%)	188 (3.0%)	
**Valvular Heart Disease**			0.356
No	5428 (87.7%)	5394 (87.2%)	
Yes	758 (12.3%)	792 (12.8%)	
**Hypertension (uncomplicated)**			0.539
No	2263 (36.6%)	2296 (37.1%)	
Yes	3923 (63.4%)	3890 (62.9%)	
**Hypertension (complications)**			0.811
No	4421 (71.5%)	4409 (71.3%)	
Yes	1765 (28.5%)	1777 (28.7%)	
**Diabetes (uncomplicated)**			0.607
No	4374 (70.7%)	4400 (71.1%)	

	White (N = 6186)	Black (N = 6186)	p-value
Yes	1812 (29.3%)	1786 (28.9%)	
**Diabetes (complications)**			0.890
No	5037 (81.4%)	5043 (81.5%)	
Yes	1149 (18.6%)	1143 (18.5%)	
**Kidney Disease**			0.407
None	5059 (81.8%)	5027 (81.3%)	
Renal Dysfunction	78 (1.3%)	67 (1.1%)	
Renal Failure	1049 (17.0%)	1092 (17.7%)	
**Liver disease**			0.360
No	6105 (98.7%)	6093 (98.5%)	
Yes	81 (1.3%)	93 (1.5%)	
**Chronic Lung Disease**			0.948
No	4836 (78.2%)	4839 (78.2%)	
Yes	1350 (21.8%)	1347 (21.8%)	
**Dementia**			0.699
No	5665 (91.6%)	5653 (91.4%)	
Yes	521 (8.4%)	533 (8.6%)	
**Ischemic event**			0.784
No	6012 (97.2%)	6017 (97.3%)	
Yes	174 (2.8%)	169 (2.7%)	
**Stroke**			0.860
No	5917 (95.7%)	5921 (95.7%)	
Yes	269 (4.3%)	265 (4.3%)	
**Hypothyroidism**			0.792
No	5132 (83.0%)	5143 (83.1%)	
Yes	1054 (17.0%)	1043 (16.9%)	
**Paralysis**			0.659
No	5922 (95.7%)	5912 (95.6%)	
Yes	264 (4.3%)	274 (4.4%)	
**Collagen Vascular Disease**			0.728
No	5825 (94.2%)	5834 (94.3%)	
Yes	361 (5.8%)	352 (5.7%)	
**Coagulopathy**			0.670
No	5826 (94.2%)	5837 (94.4%)	
Yes	360 (5.8%)	349 (5.6%)	
**Chronic Obstructive Pulmonary Disease**			0.949
No	4741 (76.6%)	4744 (76.7%)	
Yes	1445 (23.4%)	1442 (23.3%)	
**Peptic Ulcer Disease**			0.841
No	6173 (99.8%)	6174 (99.8%)	
Yes	13 (0.2%)	12 (0.2%)	
**Depression**			0.876
No	5619 (90.8%)	5624 (90.9%)	
Yes	567 (9.2%)	562 (9.1%)	
**Cushing's Disease**			0.655

	White (N = 6186)	Black (N = 6186)	p-value
No	* (100%)	* (100%)	
Yes	* (0%)	* (0%)	
**Graves Disease**			.
No	6186 (100.0%)	6186 (100.0%)	
**Post-Inflammatory Pulmonary Fibrosis**			0.597
No	6124 (99.0%)	6118 (98.9%)	
Yes	62 (1.0%)	68 (1.1%)	
**Any Breast Conserving Therapy**			0.390
No BCT	5861 (94.7%)	5882 (95.1%)	
BCT	325 (5.3%)	304 (4.9%)	
**Any Mastectomy**			0.829
No Mastectomy	1377 (22.3%)	1387 (22.4%)	
Mastectomy	4809 (77.7%)	4799 (77.6%)	
**Any Radiation**			0.815
No	3338 (54.0%)	3351 (54.2%)	
Yes	2848 (46.0%)	2835 (45.8%)	
**Any Chemotherapy**			0.967
No	4584 (74.1%)	4586 (74.1%)	
Yes	1602 (25.9%)	1600 (25.9%)	
**Any Doxorubicin**			0.797
No	5652 (91.4%)	5660 (91.5%)	
Yes	534 (8.6%)	526 (8.5%)	
**Any Taxane**			0.603
No	5416 (87.6%)	5435 (87.9%)	
Yes	770 (12.4%)	751 (12.1%)	
**Doxorubicin and BCT**			0.738
No	6144 (99.3%)	6147 (99.4%)	
Yes	42 (0.7%)	39 (0.6%)	
**Doxorubicin and Mastectomy**			0.666
No	5765 (93.2%)	5777 (93.4%)	
Yes	421 (6.8%)	409 (6.6%)	
**Doxorubicin and Radiation**			0.558
No	5879 (95.0%)	5893 (95.3%)	
Yes	307 (5.0%)	293 (4.7%)	
**Doxorubicin and Taxane**			0.632
No	5849 (94.6%)	5861 (94.7%)	
Yes	337 (5.4%)	325 (5.3%)	
**Taxane and BCT**			0.167
No	6142 (99.3%)	6154 (99.5%)	
Yes	44 (0.7%)	32 (0.5%)	
**Taxane and Mastectomy**			0.519
No	5591 (90.4%)	5612 (90.7%)	
Yes	595 (9.6%)	574 (9.3%)	
**Taxane and Radiation**			0.620
No	5752 (93.0%)	5766 (93.2%)	

	White (N = 6186)	Black (N = 6186)	p-value
Yes	434 (7.0%)	420 (6.8%)	
**Radiation and BCT**			0.916
No	6001 (97.0%)	5999 (97.0%)	
Yes	185 (3.0%)	187 (3.0%)	
**Radiation and Mastectomy**			0.686
No	3730 (60.3%)	3752 (60.7%)	
Yes	2456 (39.7%)	2434 (39.3%)	
**Radiation, Doxorubicin, Taxane**			0.423
No	5972 (96.5%)	5988 (96.8%)	
Yes	214 (3.5%)	198 (3.2%)	
**BCT, Doxorubicin, Taxane**			0.262
No	6163 (99.6%)	6170 (99.7%)	
Yes	23 (0.4%)	16 (0.3%)	
**BCT, Doxorubicin, Radiation**			0.792
No	6158 (99.5%)	6156 (99.5%)	
Yes	28 (0.5%)	30 (0.5%)	
**BCT, Taxane, Radiation**			0.772
No	6161 (99.6%)	6163 (99.6%)	
Yes	25 (0.4%)	23 (0.4%)	
**Mastectomy, Doxorubicin, Taxane**			0.473
No	5918 (95.7%)	5934 (95.9%)	
Yes	268 (4.3%)	252 (4.1%)	
**Mastectomy, Doxorubicin, Radiation**			0.375
No	5938 (96.0%)	5957 (96.3%)	
Yes	248 (4.0%)	229 (3.7%)	
**Mastectomy, Taxane, Radiation**			0.511
No	5823 (94.1%)	5840 (94.4%)	
Yes	363 (5.9%)	346 (5.6%)	
**Mastectomy, Doxorubicin, Taxane, Radiation**			0.291
No	6010 (97.2%)	6029 (97.5%)	
Yes	176 (2.8%)	157 (2.5%)	
**BCT, Doxorubicin, Taxane, Radiation**			0.853
No	6171 (99.8%)	6172 (99.8%)	
Yes	15 (0.2%)	14 (0.2%)	
**Any Breast Conserving Therapy (a)**			0.684
No BCT	5982 (96.7%)	5990 (96.8%)	
BCT	204 (3.3%)	196 (3.2%)	
**Any Mastectomy (a)**			0.632
No Mastectomy	1203 (19.4%)	1182 (19.1%)	
Mastectomy	4983 (80.6%)	5004 (80.9%)	
**Any Radiation (a)**			0.871
No	3278 (53.0%)	3287 (53.1%)	
Yes	2908 (47.0%)	2899 (46.9%)	
**Any Chemotherapy (a)**			0.596

	White (N = 6186)	Black (N = 6186)	p-value
No	4316 (69.8%)	4343 (70.2%)	
Yes	1870 (30.2%)	1843 (29.8%)	
**Any Doxorubicin**			0.797
No	5652 (91.4%)	5660 (91.5%)	
Yes	534 (8.6%)	526 (8.5%)	
**Any Taxane**			0.603
No	5416 (87.6%)	5435 (87.9%)	
Yes	770 (12.4%)	751 (12.1%)	
**Doxorubicin and BCT (a)**			0.862
No	6169 (99.7%)	6170 (99.7%)	
Yes	17 (0.3%)	16 (0.3%)	
**Doxorubicin and Mastectomy (a)**			0.652
No	5733 (92.7%)	5746 (92.9%)	
Yes	453 (7.3%)	440 (7.1%)	
**Doxorubicin and Radiation (a)**			0.592
No	5870 (94.9%)	5883 (95.1%)	
Yes	316 (5.1%)	303 (4.9%)	
**Doxorubicin and Taxane**			0.632
No	5849 (94.6%)	5861 (94.7%)	
Yes	337 (5.4%)	325 (5.3%)	
**Taxane and BCT (a)**			0.881
No	6164 (99.6%)	6163 (99.6%)	
Yes	22 (0.4%)	23 (0.4%)	
**Taxane and Mastectomy (a)**			0.674
No	5564 (89.9%)	5578 (90.2%)	
Yes	622 (10.1%)	608 (9.8%)	
**Taxane and Radiation (a)**			0.623
No	5743 (92.8%)	5757 (93.1%)	
Yes	443 (7.2%)	429 (6.9%)	
**Radiation and BCT (a)**			0.872
No	6109 (98.8%)	6107 (98.7%)	
Yes	77 (1.2%)	79 (1.3%)	
**Radiation and Mastectomy (a)**			0.841
No	3556 (57.5%)	3567 (57.7%)	
Yes	2630 (42.5%)	2619 (42.3%)	
**Radiation, Doxorubicin, and Taxane (a)**			0.427
No	5968 (96.5%)	5984 (96.7%)	
Yes	218 (3.5%)	202 (3.3%)	
**BCT, Doxorubicin, Taxane (a)**			0.796
No	* (100%)	* (100%)	
Yes	* (0%)	* (0%)	
**BCT, Doxorubicin, Radiation (a)**			0.808
No	* (100%)	* (100%)	
Yes	* (0%)	* (0%)	
**BCT, Taxane, Radiation (a)**			0.248

	White (N = 6186)	Black (N = 6186)	p-value
No	* (100%)	* (100%)	
Yes	* (0%)	* (0%)	
**Mastectomy, Doxorubicin, Taxane (a)**			0.434
No	5900 (95.4%)	5918 (95.7%)	
Yes	286 (4.6%)	268 (4.3%)	
**Mastectomy, Doxorubicin, Radiation (a)**			0.428
No	5907 (95.5%)	5925 (95.8%)	
Yes	279 (4.5%)	261 (4.2%)	
**Mastectomy, Taxane, Radiation (a)**			0.577
No	5793 (93.6%)	5808 (93.9%)	
Yes	393 (6.4%)	378 (6.1%)	
**Mastectomy, Doxorubicin, Taxane, Radiation (a)**			0.289
No	5993 (96.9%)	6013 (97.2%)	
Yes	193 (3.1%)	173 (2.8%)	
**BCT, Doxorubicin, Taxane, Radiation (a)**			0.705
No	* (100%)	* (100%)	
Yes	* (0%)	* (0%)	

Supplemental Table 3. Mapping of Silber et al. (2013) codes to the code with the highest Pointwise Mutual Information (PMI) statistic.

	Input Code	Code with highest PMI	PMI
BCT ICD9-P to ICD9-P	p_852	p_0405	10.49593
BCT ICD9-P to ICD9-P	p_852	p_86	10.49593
BCT ICD9-P to ICD9-P	p_8520	p_0529	6.654592
BCT ICD9-P to ICD9-P	p_8521	p_8519	3.78828
BCT ICD9-P to ICD9-P	p_8522	p_8302	4.64535
Mastectomy ICD9-P to ICD9-P	p_8536	p_8595	7.759936
Mastectomy ICD9-P to ICD9-P	p_8535	p_8570	5.779869
Mastectomy ICD9-P to ICD9-P	p_8534	p_6011	6.539767
Mastectomy ICD9-P to ICD9-P	p_8533	p_8595	6.530552
Mastectomy ICD9-P to ICD9-P	p_8523	p_403	4.085058
Mastectomy ICD9-P to ICD9-P	p_8541	p_7761	4.040477
Mastectomy ICD9-P to ICD9-P	p_8542	p_8595	5.342532
Mastectomy ICD9-P to ICD9-P	p_8543	p_8866	4.201203
Mastectomy ICD9-P to ICD9-P	p_8544	p_857	5.428429
Mastectomy ICD9-P to ICD9-P	p_8545	p_3001	7.814787
Mastectomy ICD9-P to ICD9-P	p_8546	p_4131	5.641629
Mastectomy ICD9-P to ICD9-P	p_8547	p_3493	5.085074
Mastectomy ICD9-P to ICD9-P	p_8548	p_8672	5.865797
Radiation ICD9-P to ICD9-P	p_9221	p_9229	6.045065
Radiation ICD9-P to ICD9-P	p_9222	p_8902	5.57419
Radiation ICD9-P to ICD9-P	p_9223	p_9905	2.519461
Radiation ICD9-P to ICD9-P	p_9224	p_9229	5.312501
Radiation ICD9-P to ICD9-P	p_9225	p_9324	7.102017
Radiation ICD9-P to ICD9-P	p_9226	p_9225	5.257355
Radiation ICD9-P to ICD9-P	p_9227	p_9985	6.920716
Radiation ICD9-P to ICD9-P	p_9228	p_9999	5.544888
Radiation ICD9-P to ICD9-P	p_9229	p_9224	5.344162
BCT ICD9-P to HCPCS	p_852	h_11404	5.671362
BCT ICD9-P to HCPCS	p_8520	h_64784	5.489409
BCT ICD9-P to HCPCS	p_8521	h_19120	3.108707
BCT ICD9-P to HCPCS	p_8522	h_19160	3.561968
Mastectomy ICD9-P to HCPCS	p_8536	h_15777	5.428488
Mastectomy ICD9-P to HCPCS	p_8535	h_19182	7.442487
Mastectomy ICD9-P to HCPCS	p_8534	h_19182	7.017331
Mastectomy ICD9-P to HCPCS	p_8533	h_Q0164	5.087214
Mastectomy ICD9-P to HCPCS	p_8523	h_19160	3.398192
Mastectomy ICD9-P to HCPCS	p_854	h_84233	5.589849
Mastectomy ICD9-P to HCPCS	p_8541	h_19180	4.945159
Mastectomy ICD9-P to HCPCS	p_8542	h_19180	4.831124
Mastectomy ICD9-P to HCPCS	p_8543	h_19240	4.47892

	Input Code	Code with highest PMI	PMI
Mastectomy ICD9-P to HCPCS	p_8544	h_15755	4.413265
Mastectomy ICD9-P to HCPCS	p_8545	h_19200	6.697163
Mastectomy ICD9-P to HCPCS	p_8546	h_19200	5.55368
Mastectomy ICD9-P to HCPCS	p_8547	h_19220	6.791945
Mastectomy ICD9-P to HCPCS	p_8548	h_12045	9.589702
Radiation ICD9-P to HCPCS	p_9221	h_77418	4.751666
Radiation ICD9-P to HCPCS	p_9222	h_77402	5.517993
Radiation ICD9-P to HCPCS	p_9223	h_79001	6.398317
Radiation ICD9-P to HCPCS	p_9224	h_77413	3.208934
Radiation ICD9-P to HCPCS	p_9225	h_77413	3.406914
Radiation ICD9-P to HCPCS	p_9226	h_B4152	7.141101
Radiation ICD9-P to HCPCS	p_9227	h_77777	6.196675
Radiation ICD9-P to HCPCS	p_9228	h_78120	5.585097
Radiation ICD9-P to HCPCS	p_9229	h_G0174	4.194677
BCT HCPCS to HCPCS	h_19160	h_00400	2.763163
BCT HCPCS to HCPCS	h_19162	h_00404	2.86373
BCT HCPCS to HCPCS	h_19120	h_00400	2.833977
Mastectomy HCPCS to HCPCS	h_19125	h_76096	3.017152
Mastectomy HCPCS to HCPCS	h_19126	h_19291	4.034323
Mastectomy HCPCS to HCPCS	h_19180	h_Y1023	3.243984
Mastectomy HCPCS to HCPCS	h_19182	h_15834	5.699537
Mastectomy HCPCS to HCPCS	h_19200	h_15101	5.848715
Mastectomy HCPCS to HCPCS	h_19220	h_19271	6.375456
Mastectomy HCPCS to HCPCS	h_19240	h_00404	4.270618
Mastectomy HCPCS to HCPCS	h_19260	h_00470	6.023472
Mastectomy HCPCS to HCPCS	h_19271	h_00472	8.909099
Mastectomy HCPCS to HCPCS	h_19301	h_77032	2.395462
Mastectomy HCPCS to HCPCS	h_19302	h_G8879	2.557967
Mastectomy HCPCS to HCPCS	h_19303	h_19357	3.02461
Mastectomy HCPCS to HCPCS	h_19304	h_21600	4.420458
Mastectomy HCPCS to HCPCS	h_19305	h_20101	4.431708
Mastectomy HCPCS to HCPCS	h_19306	h_69979	4.847908
Mastectomy HCPCS to HCPCS	h_19307	h_88309	3.210545
Radiation HCPCS to HCPCS	h_77261	h_77420	6.047489
Radiation HCPCS to HCPCS	h_77262	h_27355	6.28636
Radiation HCPCS to HCPCS	h_77263	h_77290	3.743933
Radiation HCPCS to HCPCS	h_77280	h_77786	4.198862
Radiation HCPCS to HCPCS	h_77285	h_77262	5.470546
Radiation HCPCS to HCPCS	h_77290	h_77263	3.449288
Radiation HCPCS to HCPCS	h_77295	h_77334	3.499339
Radiation HCPCS to HCPCS	h_77299	h_76815	5.479562
Radiation HCPCS to HCPCS	h_77300	h_77786	3.532129

	Input Code	Code with highest PMI	PMI
Radiation HCPCS to HCPCS	h_77301	h_77338	5.385918
Radiation HCPCS to HCPCS	h_77305	h_77415	3.985497
Radiation HCPCS to HCPCS	h_77310	h_77262	4.922503
Radiation HCPCS to HCPCS	h_77315	h_77334	3.440289
Radiation HCPCS to HCPCS	h_77321	h_77427	3.222923
Radiation HCPCS to HCPCS	h_77331	h_77413	3.191288
Radiation HCPCS to HCPCS	h_77332	h_77263	3.406297
Radiation HCPCS to HCPCS	h_77333	h_77310	3.802176
Radiation HCPCS to HCPCS	h_77334	h_77300	3.350468
Radiation HCPCS to HCPCS	h_77336	h_77413	3.456563
Radiation HCPCS to HCPCS	h_77338	h_77301	5.584335
Radiation HCPCS to HCPCS	h_77370	h_Q3001	4.692803
Radiation HCPCS to HCPCS	h_77371	h_61796	8.74476
Radiation HCPCS to HCPCS	h_77372	h_61796	9.334266
Radiation HCPCS to HCPCS	h_77373	h_77435	9.439569
Radiation HCPCS to HCPCS	h_77399	h_77366	7.65047
Radiation HCPCS to HCPCS	h_77400	h_77405	10.5637
Radiation HCPCS to HCPCS	h_77401	h_77405	6.126816
Radiation HCPCS to HCPCS	h_77402	h_77420	6.496469
Radiation HCPCS to HCPCS	h_77403	h_77420	5.214699
Radiation HCPCS to HCPCS	h_77404	h_Q0040	4.692691
Radiation HCPCS to HCPCS	h_77405	h_77400	10.61825
Radiation HCPCS to HCPCS	h_77406	h_77420	5.309888
Radiation HCPCS to HCPCS	h_77407	h_77425	6.246562
Radiation HCPCS to HCPCS	h_77408	h_77425	5.212277
Radiation HCPCS to HCPCS	h_77409	h_77499	5.037132
Radiation HCPCS to HCPCS	h_77410	h_77405	9.470787
Radiation HCPCS to HCPCS	h_77411	h_77410	6.750286
Radiation HCPCS to HCPCS	h_77412	h_77430	3.822135
Radiation HCPCS to HCPCS	h_77413	h_77336	3.228362
Radiation HCPCS to HCPCS	h_77414	h_77336	3.129313
Radiation HCPCS to HCPCS	h_77415	h_90620	8.082694
Radiation HCPCS to HCPCS	h_77416	h_77336	3.072726
Radiation HCPCS to HCPCS	h_77417	h_77413	3.419607
Radiation HCPCS to HCPCS	h_77418	h_77301	4.030586
Radiation HCPCS to HCPCS	h_77419	h_77431	4.366374
Radiation HCPCS to HCPCS	h_77421	h_77418	3.964204
Radiation HCPCS to HCPCS	h_77427	h_77413	3.10682
Radiation HCPCS to HCPCS	h_77435	h_63620	9.808979
Radiation HCPCS to HCPCS	h_77470	h_77781	3.591988
Radiation HCPCS to HCPCS	h_77499	h_77409	5.430983
Radiation HCPCS to HCPCS	h_77600	h_77402	5.668005

	Input Code	Code with highest PMI	PMI
Radiation HCPCS to HCPCS	h_77605	h_85095	5.450874
Radiation HCPCS to HCPCS	h_77610	h_30801	7.247396
Radiation HCPCS to HCPCS	h_77615	h_77778	6.738606
Radiation HCPCS to HCPCS	h_77750	h_A9605	9.282598
Radiation HCPCS to HCPCS	h_77761	h_79300	7.356399
Radiation HCPCS to HCPCS	h_77762	h_01900	9.064779
Radiation HCPCS to HCPCS	h_77763	h_77762	6.750475
Radiation HCPCS to HCPCS	h_77776	h_77781	5.371496
Radiation HCPCS to HCPCS	h_77777	h_78799	8.009155
Radiation HCPCS to HCPCS	h_77778	h_77790	7.197975
Radiation HCPCS to HCPCS	h_77789	h_67218	8.805457
Radiation HCPCS to HCPCS	h_77790	h_77778	7.056798
Radiation HCPCS to HCPCS	h_G0173	h_G0338	9.218512
Radiation HCPCS to HCPCS	h_G0174	h_G0178	7.854102
Radiation HCPCS to HCPCS	h_G0178	h_G0174	8.559884
Radiation HCPCS to HCPCS	h_G0242	h_G0243	10.25485
Radiation HCPCS to HCPCS	h_G0243	h_G0242	10.27381
Radiation HCPCS to HCPCS	h_G0251	h_63620	7.868359
Radiation HCPCS to HCPCS	h_G0338	h_G0173	9.747983
Radiation HCPCS to HCPCS	h_G0339	h_G0340	9.007956
Radiation HCPCS to HCPCS	h_G0340	h_G0339	8.79126
Radiation HCPCS to HCPCS	h_61793	h_G0243	8.610794
Radiation HCPCS to HCPCS	h_G8378	h_G8381	6.844016
Radiation HCPCS to HCPCS	h_G8379	h_G8381	6.595152
Radiation HCPCS to HCPCS	h_C9726	h_77424	5.473863
Radiation HCPCS to HCPCS	h_C9728	h_49411	5.688734
Radiation HCPCS to HCPCS	h_A4650	h_64435	6.99525
Chemotherapy HCPCS to HCPCS	h_C1167	h_C9119	4.742848
Chemotherapy HCPCS to HCPCS	h_C9115	h_Q0081	4.360678
Chemotherapy HCPCS to HCPCS	h_C9120	h_C1774	6.497725
Chemotherapy HCPCS to HCPCS	h_C9127	h_Q0137	5.473291
Chemotherapy HCPCS to HCPCS	h_C9399	h_44020	4.807499
Chemotherapy HCPCS to HCPCS	h_C9411	h_90781	6.350283
Chemotherapy HCPCS to HCPCS	h_C9415	h_C9421	6.269753
Chemotherapy HCPCS to HCPCS	h_C9420	h_C9415	5.998119
Chemotherapy HCPCS to HCPCS	h_C9421	h_C9415	6.064197
Chemotherapy HCPCS to HCPCS	h_C9431	h_Q0137	4.170191
Chemotherapy HCPCS to HCPCS	h_C9432	h_38724	5.503255
Chemotherapy HCPCS to HCPCS	h_G0356	h_J9395	6.773325
Chemotherapy HCPCS to HCPCS	h_G8371	h_G8374	9.764241
Chemotherapy HCPCS to HCPCS	h_G8373	h_G8381	5.392053
Chemotherapy HCPCS to HCPCS	h_G8374	h_G8371	9.265043

	Input Code	Code with highest PMI	PMI
Chemotherapy HCPCS to HCPCS	h_J0207	h_J9062	4.142484
Chemotherapy HCPCS to HCPCS	h_J0640	h_J9263	4.609867
Chemotherapy HCPCS to HCPCS	h_J1950	h_A4246	8.762833
Chemotherapy HCPCS to HCPCS	h_J7150	h_J9200	8.825699
Chemotherapy HCPCS to HCPCS	h_J8520	h_J8521	7.721685
Chemotherapy HCPCS to HCPCS	h_J8521	h_J8520	8.039825
Chemotherapy HCPCS to HCPCS	h_J8530	h_Q0173	4.92821
Chemotherapy HCPCS to HCPCS	h_J8610	h_E0500	7.715521
Chemotherapy HCPCS to HCPCS	h_J8999	h_J8521	6.449461
Chemotherapy HCPCS to HCPCS	h_J9000	h_J9093	3.071157
Chemotherapy HCPCS to HCPCS	h_J9001	h_J9041	4.088734
Chemotherapy HCPCS to HCPCS	h_J9035	h_67028	5.900116
Chemotherapy HCPCS to HCPCS	h_J9045	h_96417	3.140661
Chemotherapy HCPCS to HCPCS	h_J9070	h_J9000	3.024771
Chemotherapy HCPCS to HCPCS	h_J9080	h_J2995	4.889798
Chemotherapy HCPCS to HCPCS	h_J9090	h_K0284	4.91385
Chemotherapy HCPCS to HCPCS	h_J9093	h_96408	3.304308
Chemotherapy HCPCS to HCPCS	h_J9094	h_J9200	4.870586
Chemotherapy HCPCS to HCPCS	h_J9095	h_J9094	4.552794
Chemotherapy HCPCS to HCPCS	h_J9096	h_WG759	4.109844
Chemotherapy HCPCS to HCPCS	h_J9097	h_80088	4.103072
Chemotherapy HCPCS to HCPCS	h_J9170	h_90767	2.946404
Chemotherapy HCPCS to HCPCS	h_J9178	h_96411	3.453771
Chemotherapy HCPCS to HCPCS	h_J9180	h_96408	4.001491
Chemotherapy HCPCS to HCPCS	h_J9190	h_J9260	4.226303
Chemotherapy HCPCS to HCPCS	h_J9200	h_J7150	8.275206
Chemotherapy HCPCS to HCPCS	h_J9202	h_J9217	5.994776
Chemotherapy HCPCS to HCPCS	h_J9217	h_96402	6.784275
Chemotherapy HCPCS to HCPCS	h_J9218	h_85244	7.052136
Chemotherapy HCPCS to HCPCS	h_J9250	h_J9190	4.134011
Chemotherapy HCPCS to HCPCS	h_J9260	h_J9190	4.416338
Chemotherapy HCPCS to HCPCS	h_J9264	h_J9035	3.844866
Chemotherapy HCPCS to HCPCS	h_J9265	h_J1200	3.123966
Chemotherapy HCPCS to HCPCS	h_J9280	h_J9340	7.099844
Chemotherapy HCPCS to HCPCS	h_J9290	h_51720	7.787488
Chemotherapy HCPCS to HCPCS	h_J9291	h_51720	9.416453
Chemotherapy HCPCS to HCPCS	h_J9293	h_J9340	6.036588
Chemotherapy HCPCS to HCPCS	h_J9355	h_96413	3.111395
Chemotherapy HCPCS to HCPCS	h_J9390	h_96409	3.747106
Chemotherapy HCPCS to HCPCS	h_J9395	h_96402	6.6491
Chemotherapy HCPCS to HCPCS	h_J9999	h_J1830	4.366339
Chemotherapy HCPCS to HCPCS	h_C8953	h_C8954	4.860028

	Input Code	Code with highest PMI	PMI
Chemotherapy HCPCS to HCPCS	h_C8954	h_C8955	5.042335
Chemotherapy HCPCS to HCPCS	h_C8955	h_C8954	5.243729
Chemotherapy HCPCS to HCPCS	h_G0355	h_G0346	4.488552
Chemotherapy HCPCS to HCPCS	h_G0359	h_G9021	4.880093
Chemotherapy HCPCS to HCPCS	h_G0361	h_J9263	5.603114
Chemotherapy HCPCS to HCPCS	h_G8371	h_G8374	9.764241
Chemotherapy HCPCS to HCPCS	h_G8374	h_G8371	9.265043
Chemotherapy HCPCS to HCPCS	h_Q0081	h_C9124	3.742458
Chemotherapy HCPCS to HCPCS	h_Q0083	h_J9218	4.470806
Chemotherapy HCPCS to HCPCS	h_Q0084	h_J9218	4.157278
Chemotherapy HCPCS to HCPCS	h_Q0085	h_C1167	4.096651
Mastectomy HCPCS to ICD9-P	h_19125	p_8521	3.199102
Mastectomy HCPCS to ICD9-P	h_19126	p_1991	3.814511
Mastectomy HCPCS to ICD9-P	h_19180	p_8541	5.201095
Mastectomy HCPCS to ICD9-P	h_19182	p_8534	7.401465
Mastectomy HCPCS to ICD9-P	h_19200	p_8545	6.918666
Mastectomy HCPCS to ICD9-P	h_19220	p_8547	6.994375
Mastectomy HCPCS to ICD9-P	h_19240	p_8543	4.763079
Mastectomy HCPCS to ICD9-P	h_19260	p_344	7.388409
Mastectomy HCPCS to ICD9-P	h_19271	p_3440	10.13629
Mastectomy HCPCS to ICD9-P	h_19303	p_8579	2.941846
Mastectomy HCPCS to ICD9-P	h_19304	p_8536	4.463643
Mastectomy HCPCS to ICD9-P	h_19305	p_8575	4.106875
Mastectomy HCPCS to ICD9-P	h_19306	p_3493	6.071565
Mastectomy HCPCS to ICD9-P	h_19307	p_8544	2.256683
BCT HCPCS to ICD9-P	h_19160	p_8523	3.759341
BCT HCPCS to ICD9-P	h_19162	p_403	4.071062
BCT HCPCS to ICD9-P	h_19120	p_8521	3.649082
Radiation HCPCS to ICD9-P	h_77261	p_7771	2.935805
Radiation HCPCS to ICD9-P	h_77262	p_8155	4.178008
Radiation HCPCS to ICD9-P	h_77263	p_X922	2.827055
Radiation HCPCS to ICD9-P	h_77280	p_9221	1.848078
Radiation HCPCS to ICD9-P	h_77285	p_9229	3.857582
Radiation HCPCS to ICD9-P	h_77290	p_X922	2.362587
Radiation HCPCS to ICD9-P	h_77295	p_9231	2.292591
Radiation HCPCS to ICD9-P	h_77299	p_8838	3.028897
Radiation HCPCS to ICD9-P	h_77300	p_9229	2.257135
Radiation HCPCS to ICD9-P	h_77301	p_3829	2.661378
Radiation HCPCS to ICD9-P	h_77305	p_9229	2.980836
Radiation HCPCS to ICD9-P	h_77310	p_9229	3.263155
Radiation HCPCS to ICD9-P	h_77315	p_X922	3.434952
Radiation HCPCS to ICD9-P	h_77321	p_9229	2.692537

	Input Code	Code with highest PMI	PMI
Radiation HCPCS to ICD9-P	h_77331	p_9229	2.17456
Radiation HCPCS to ICD9-P	h_77332	p_X922	3.354314
Radiation HCPCS to ICD9-P	h_77333	p_8908	3.752464
Radiation HCPCS to ICD9-P	h_77334	p_9229	2.238354
Radiation HCPCS to ICD9-P	h_77336	p_9229	2.356678
Radiation HCPCS to ICD9-P	h_77370	p_9231	3.00757
Radiation HCPCS to ICD9-P	h_77399	p_6991	5.72411
Radiation HCPCS to ICD9-P	h_77401	p_9222	3.63948
Radiation HCPCS to ICD9-P	h_77402	p_9222	5.156141
Radiation HCPCS to ICD9-P	h_77403	p_8827	3.252794
Radiation HCPCS to ICD9-P	h_77404	p_8743	2.685666
Radiation HCPCS to ICD9-P	h_77406	p_8606	3.907662
Radiation HCPCS to ICD9-P	h_77407	p_X922	4.65225
Radiation HCPCS to ICD9-P	h_77408	p_9229	3.418839
Radiation HCPCS to ICD9-P	h_77409	p_9229	1.456448
Radiation HCPCS to ICD9-P	h_77411	p_8879	3.594156
Radiation HCPCS to ICD9-P	h_77412	p_9229	3.633756
Radiation HCPCS to ICD9-P	h_77413	p_9229	2.668953
Radiation HCPCS to ICD9-P	h_77414	p_9223	2.535324
Radiation HCPCS to ICD9-P	h_77416	p_9229	2.41967
Radiation HCPCS to ICD9-P	h_77417	p_9229	2.466664
Radiation HCPCS to ICD9-P	h_77418	p_3829	2.307659
Radiation HCPCS to ICD9-P	h_77419	p_9749	2.453114
Radiation HCPCS to ICD9-P	h_77427	p_9223	2.597482
Radiation HCPCS to ICD9-P	h_77470	p_9231	2.109786
Radiation HCPCS to ICD9-P	h_77499	p_9229	3.015592
Radiation HCPCS to ICD9-P	h_77615	p_9924	6.643745
Radiation HCPCS to ICD9-P	h_77750	p_9227	4.585632
Radiation HCPCS to ICD9-P	h_77761	p_6991	4.542398
Radiation HCPCS to ICD9-P	h_77762	p_6995	8.42582
Radiation HCPCS to ICD9-P	h_77763	p_6995	5.618951
Radiation HCPCS to ICD9-P	h_77776	p_9227	5.68967
Radiation HCPCS to ICD9-P	h_77777	p_9227	7.373596
Radiation HCPCS to ICD9-P	h_77778	p_9227	6.439969
Radiation HCPCS to ICD9-P	h_77789	p_9227	2.380943
Radiation HCPCS to ICD9-P	h_77790	p_6991	5.318712
Radiation HCPCS to ICD9-P	h_G0173	p_9232	8.795099
Radiation HCPCS to ICD9-P	h_G0174	p_9229	5.239349
Radiation HCPCS to ICD9-P	h_G0178	p_9229	3.900418
Radiation HCPCS to ICD9-P	h_G0242	p_G024	10.67677
Radiation HCPCS to ICD9-P	h_G0243	p_G024	10.75261
Radiation HCPCS to ICD9-P	h_61793	p_9232	9.003593

	Input Code	Code with highest PMI	PMI
Radiation HCPCS to ICD9-P	h_G8379	p_8395	2.2528
